# Reconsidering first-line treatment for obstructive sleep apnea: a systematic review of the literature

**DOI:** 10.1186/s40463-016-0136-4

**Published:** 2016-04-06

**Authors:** Brian W. Rotenberg, Claudio Vicini, Edward B. Pang, Kenny P. Pang

**Affiliations:** Department of Otolaryngology–Head and Neck Surgery, Western University, London, Ontario Canada; Head & Neck Department, ASL of Romagna, ENT and Oral Surgery Unit, Morgagni-Pierantoni Hospital (Forlì), Ospedale degli Infermi (Faenza), Forlì, Italy; Asia Sleep Centre, Paragon, Singapore; St. Joseph’s Hospital, Room B2-501, 268 Grosvenor Street, London, ON N6A 4V2 Canada

**Keywords:** Obstructive sleep apnea, Uvulopalatoplasty, CPAP

## Abstract

**Background:**

Continuous positive airway pressure (CPAP) is typically recommended as first line therapy for obstructive sleep apnea, but the adherence rate of CPAP is problematic. This study’s objective was to systematically review the literature relating to CPAP as first line therapy for OSA and compare it to surgical literature on the same topic.

**Methods:**

A systematic review was conducted according to PRISMA guidelines, examining Medline-Ovid, Embase, and Pubmed databases. The primary search objective was to identify all papers reporting the results of (1) randomized clinical trials (RCT) of CPAP for the treatment of adults with OSA; and (2) both randomized and non-randomized clinical trials and case series on the surgical treatment of OSA in adults. A PhD-level biostatistician first screened papers, and then those that met study criteria were retrieved and analyzed using standardized forms for each author. The primary outcomes were adherence rates of CPAP.

**Results:**

A total of 82 controlled clinical trials for CPAP and 69 controlled and non-controlled surgery trials were identified for analysis. Variation in CPAP use within reported RCT trials were identified, and the majority of patients in the studies would eventually be considered non-adherent to CPAP.

**Conclusions:**

When considering the numerous patient-related factors that come into play when CPAP is prescribed, the concept of CPAP as gold-standard therapy for OSA should be reconsidered. In many cases surgery can provide a better overall outcome. This study’s results suggest that certain patients with OSA may be managed more effectively with surgery than CPAP, without confounding issues of treatment adherence.

**Electronic supplementary material:**

The online version of this article (doi:10.1186/s40463-016-0136-4) contains supplementary material, which is available to authorized users.

## Background

Obstructive sleep apnea (OSA) is considered part of a group of disorders that cover a continuum ranging from habitual snoring (simple snoring) to moderate or severe OSAS. OSA is characterized by repetitive apnea and/or hypopnea during sleep. Due to relaxation of the upper airway pharyngeal and tongue muscles during sleep the airway narrows and collapses resulting in hypoxaemia, increased sympathetic overdrive, increased blood pressure, and hypercapnia. These add hypoxic stress to the brain and heart. Apneic and hypopneic events may occur numerous times per night, resulting in arousals from sleep and sleep disruptions causing sleep fragmentation leading to excessive daytime sleepiness. These repeated cyclic oxygen desaturations and a fragmented sleep architecture lead to sympathetic overdrive, interrupted sleep, and reduced percentage of slow wave sleep, translating into symptoms of daytime somnolence, morning headaches, poor concentration, memory loss, a higher risk of car accidents, depression and marital discord.

OSA has a strong association with hypertension, atherosclerosis, and cerebrovascular accidents (strokes) [1]. Studies have also shown a higher mortality rate among patients with cardiovascular disease who also have OSA [1]. It has been long purported that nasal continuous positive airway pressure (CPAP) is the “gold” standard in the treatment of OSA, and there is no doubt that CPAP is effective when used properly and according to AASM standards. However, it is also well known that due to problematic patient adherence, the real world effectiveness of CPAP is low, with a large proportion of users abandoning the machine within one year of prescription. Such patients cannot be said to be effectively treated. Surgery for OSA on the other hand does not rely on any form of long-term patient adherence, and when the right patient is matched with the right pharyngeal procedure in order to maximize success, long-term strong results have been shown. When considering all OSA patients it is recognized that overall treatment success rates with surgery are lower than via CPAP, but this does not hold true for the subset of patients with appropriate apnea-specific surgical anatomy wherein rates of successful surgical OSA treatment are very high. Moreover the issue of CPAP adherence has generally not been examined during these debates; to make an effective comparison adherence must be taken into account when studying the impact on OSA of CPAP versus surgery. CPAP, an efficacious therapy with inconsistent adherence, can potentially be equivalent to surgery, that being a “partial” therapy with complete adherence. It is the issue of treatment effectiveness versus adherence (the relationship of the two defining success) that is at the crux of the matter.

This study’s objective was to systematically review the literature relating to CPAP as first line therapy for OSA and then compare it to surgical literature on the same topic.

## Methods

Our review was carried out in accordance with the preferred reporting items for systematic review and meta-analysis protocols (PRISMA-P) 2015 statement. A comprehensive systematic literature review was conducted using the Medline-Ovid, Embase, and Pubmed databases.

The primary search objective was to identify all papers reporting the results of (1) randomized clinical trials (RCT) of CPAP for the treatment of adults with OSA; and (2) both randomized and non-randomized clinical trials and case series on the surgical treatment of OSA in adults. The first step was a locate and review all of the studies listed for analysis in three major literature reviews, a Cochrane Collaboration review [1] and a second systematic literature review published by the National Institutes of Health Research (NIHR) [2] on the use of CPAP for the treatment of OSA, and a second Cochrane Collaboration review on its surgical management [3]. The second step was an extensive search of the PubMed/MedLine database, initiated using the following combined search terms: “randomized clinical trial and obstructive sleep apnea” (*n* = 1083); “CPAP and randomized clinical trial and obstructive sleep apnea” (*n* = 357); and “surgery and obstructive sleep apnea and clinical trial” (*n* = 603). From these lists, studies were identified that (a) did not replicate studies already found and (b) were otherwise eligible for inclusion. The third and final step was a review of all reference lists and tables of other studies found within papers identified in the second step. A PhD level biostatistician performed all three of the search steps.

Articles were considered for inclusion into the study by reviewing the titles and abstracts of all retrieved studies. The senior study authors BWR and KPP did this and results were compiled to ensure no studies were missed. The full text of selected studies were then analyzed to ensure that the following inclusion criteria were met: diagnosis of obstructive sleep apnea, no confounding data for central sleep apnea, and the paper referred to either CPAP or surgical treatment of OSA.

## Results

**Fig. 1:**
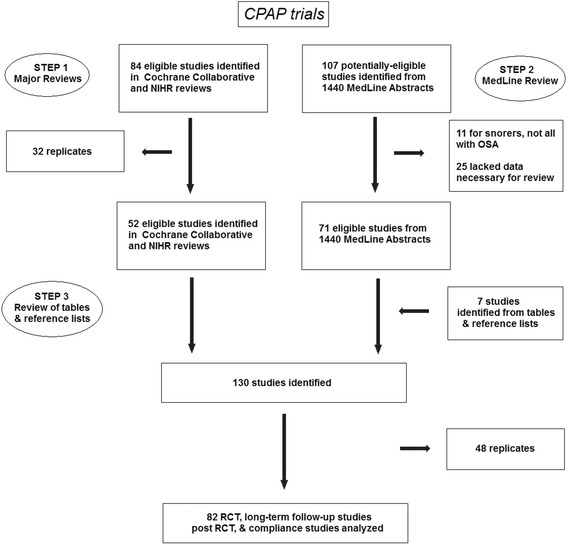
PRISMA chart of CPAP study search strategy

**Fig. 2:**
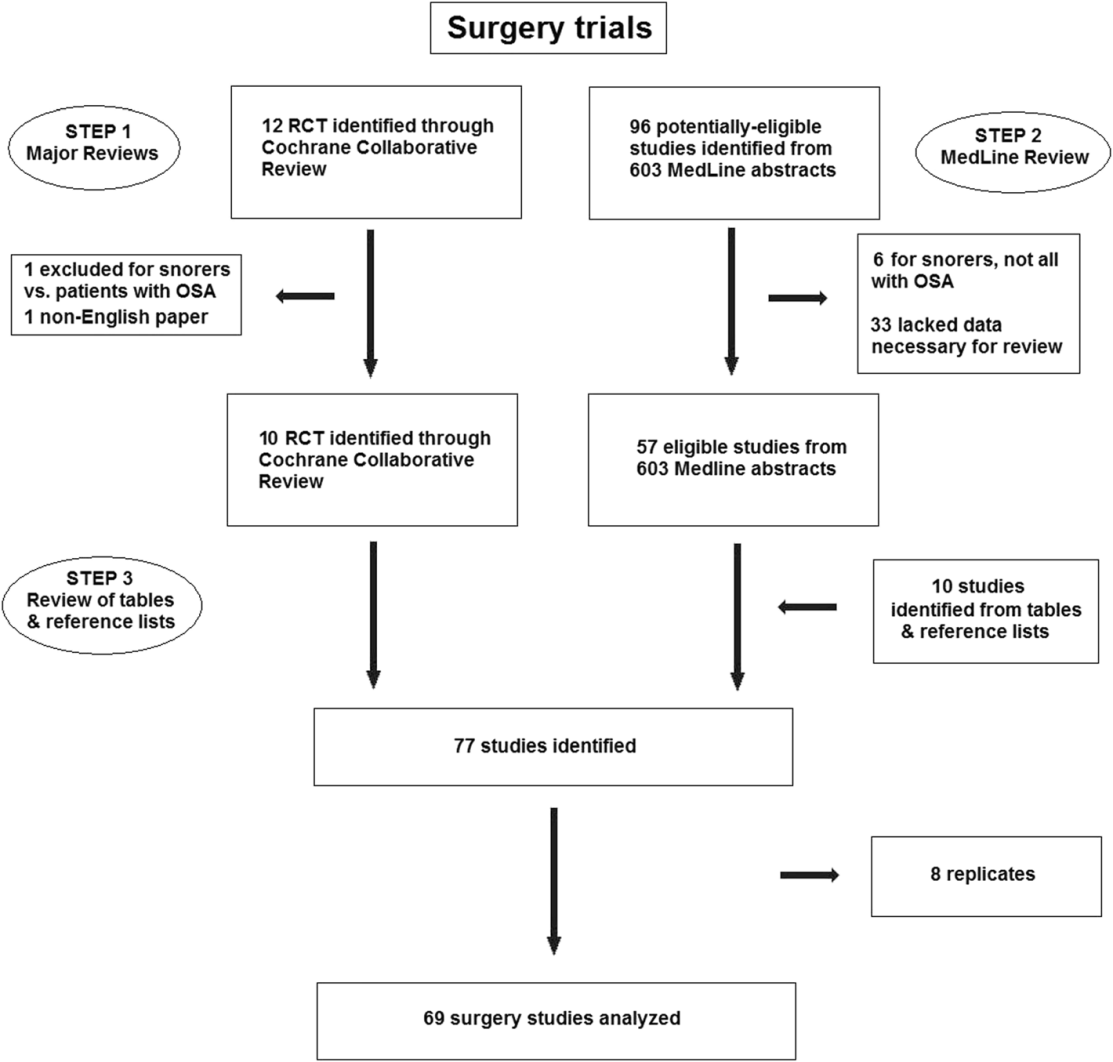
PRISMA chart of surgery study search strategy

A total of 82 controlled clinical trials for CPAP and 69 controlled and non-controlled surgery trials were identified for analysis (note that non-controlled trials were accepted for surgery because of the relatively few controlled trials). The CPAP studies included trials comparing CPAP versus sub-therapeutic (sham) CPAP [4–34], CPAP versus an oral placebo [33, 35–43], CPAP versus conservative or no therapy [10, 22, 44–54], CPAP versus an oral appliance [4, 5, 36, 51, 55–63], CPAP versus postural therapy [64–67], and CPAP alone assessing different means to modify adherence (e.g., with vs. without a humidifying element) [8, 21, 30, 68–79]. The surgical trials assessed a variety of single- and multi-stage procedures incorporating uvuloplasty [22, 80–117], mandibular advancement [118, 119], laser treatments [120–125], radiofrequency tissue reduction and other lingual procedures [117, 125–134], and palate implants [135–141], in addition to five trials specifically evaluating the safety versus risks of OSA surgery, including its safety as an outpatient/same-day procedure [142–146]. The PRISMA charts seen in Figs. 1 and 2 summarize the study flow, and Additional files 1, 2, and Table 1 summarize the results of the search strategy.

**Table 1:** CPAP versus surgery comparisons

First author - year	EBM rating	Study design	Treatment groups	Study findings	Study limitations/issues
Woodson 2003	1	RCT	nCPAP vs. RFTR vs. sham RFTR	Relative to sham Rx, rxn time & fastest rxn time both improved post-RFTR (*p* = 0.03 & 0.02) but not on CPAP.	Very poor CPAP complance (~16 h/week);
				ESS ↓ similarly with RFTR & CPAP (−2.1 vs. −2.3, *p* = 0.005 & 0.02). SNORE25 score ↓ w/both (*p* < 0.001 vs. 0.005)	Different # of Rx sessions in RFTR (4.5) vs. sham RFTR (2.9) groups
Ceylan 2009	3	nonRCT	TC-RFTR vs. nCPAP	Both RFTR & CPAP → ↓AHI (28.5 → 15.7 vs 29.6 → 16.1, both *p* < 0.001; NS); ↓ESS (11.1 → 8.4, *p* = 0.003 vs 10.8 → 8.2, *p* = 0.003; NS);	Non-random allocation to Rx/potential selection bias;
				↓CT90 (15.2 → 11.1 % vs 14.3 → 10.7 %, both *p* < 0.001; NS); & ↑LSAT (88.4 → 93.5, *p* = 0.03 vs 86.8 → 94.6 %, *p* < 0.001, NS). 53.8 vs. 52.4 % responders	Compliance with CPAP not reported
Weaver 2004	1	pop. survey	UPPP ± TE ± SP ± other vs. CPAP	1339/18,754 (7.1 %) died w/ CPAP vs. 71/2072 (3.4 %) post-op. Adjusting for age, gender, race, year of Rx & co-morbidities,	Retrospective analysis; potential confounders missing
				MR ↑ 31 % (95 % CI 3–67 %) w/CPAP (*p* = 0.03)	(e.g., severity of OSA, overall health status)

## Discussion of findings

Currently, continuous positive airway pressure (CPAP) is considered the gold standard treatment for patients with obstructive sleep apnea, be it mild, moderate or severe. This is the conclusion expressed in both a recently-published Cochrane Collaboration review [1] and a second systematic literature review published by the National Institutes of Health Research (NIHR) [2]. Surgical approaches are hardly discussed at all in either of these two reviews, largely on the basis of the lack of randomized controlled trials (RCTs). A closer look at the evidence however reveals that surgery may indeed play a primary role in many patients with OSA.

First, although a large number of RCTs have been published documenting the benefits of CPAP relative to sub-therapeutic (sham) CPAP [3–33], an oral placebo [32, 34–43], conservative or no therapy [9, 21, 42, 44–53], various oral appliances [3, 4, 35, 50, 54–62], and postural therapy [63–66], numerous limitations of these RCTs must be considered. First among these is the short duration of follow-up that has been almost ubiquitous amongst CPAP trials, the vast majority having final assessments within weeks of the initial treatment, and only a small handful extending beyond 3 to 4 months [46, 56], 6 months [3, 52], 1 year [9, 21], or beyond [44]. A couple of additional long-term cohort studies emerged from RCT, following patients, open label, to and beyond 1 year [3, 62]. This contrasts, however, with the much more long-term follow-up generally performed for surgical trials, where follow-up to and beyond 6 months is the norm, with several investigative groups reporting on outcomes beyond 1 year.

A second issue pertains to adherence with CPAP, a well-documented problem that warrants concern. In our review of 83 CPAP trials (Additional file 1), the average patient in bed for 7 h across these 83 closely supervised clinical trials was not using it an average of 32.9 % of the time. When the nights per week of CPAP non-use have been examined, the percentages range from 10 to 40 % [5, 35, 50, 55, 67–70] These are problematic percentages given that several published RCT have documented that at least a minimum level of CPAP use of 5–6 h per night is required to reap benefits from it [9, 37, 40, 68, 71]. There is therefore a sizeable subset of patients on CPAP who either cease to use it altogether, or fail to use it enough hours per night and/or nights per week to achieve clinically-significant benefits.

The issue of adherence is generally a non-issue with surgery, especially beyond the initial recovery period. Once a patient’s anatomy is changed, it should remain so. How effective is surgery? Admittedly, there are far fewer EBM (evidence-based medicine) level 1 RCT and many more EBM level 4 case series for OSA surgery, as should be expected given the ethical and methodological obstacles associated with performing double-blinded or even controlled surgical trials. This being said, only 24 of the CPAP RCT described above were truly blinded, pitting therapeutic CPAP against sub-therapeutic (and thereby, sham) CPAP, rendering all comparisons, especially of subjective measures like the patient’s level of sleepiness and quality of life, at least somewhat suspect. Clearly, all subjects in these studies knew that they were using an oral appliance, an oral placebo or nothing versus nasal CPAP. Interestingly, and harkening back to the issue of adherence, though CPAP tended to improve objective measures of OSA to a greater degree than oral appliances, patients consistently and decidedly preferred the latter [35, 55, 57, 60].

The limitations of EBM level 4 evidence set aside, among the 1802 patients who underwent OSA surgery across the 53 studies we analyzed, more than half (957, 53.1 %) were deemed to have experienced a ‘good response’. This is despite the consistent use of strict criteria for a ‘response’ that ranged from Sher’s criteria of no less than a 50 % reduction in AHI to a level of 20 events/hour or less [79], criteria that were used in 20 of the studies [80–99]; to being as stringent as no less than a 50 % reduction in RDI (which is similar to the AHI but also incorporates near-hyponeic events) to a level of 20 events/hour or less [100–106]; no less than a 50 % reduction in either AHI or RDI (the latter also incorporating near-hyponeic events) to a level of 15 or even 10 events/hour or less [91, 107–110]; and reducing AHI or RDI to ≤10 or even 5 events/hour [111–114] (Additional file 2). Mandibular/maxillo-mandibular advancement procedures had an especially high success (good response) rate of 87.0 % [84, 107, 115]. Moreover, there appeared to be a dose–response effect, with more aggressive procedures incorporating UPPP more effective than less aggressive procedures like tongue tissue ablation (Additional file 2), and more repetitions of treatments like laser- or radiofrequency- aided tissue reduction more effective than fewer repetitions [116].

Only three studies directly have compared CPAP and OSA surgery: one a randomized clinical trial comparing therapeutic radiofrequency-aided tissue reduction (RFTR), sham RFTR and nasal CPAP [117]; one a non-randomized clinical trial comparing RFTR and nasal CPAP [83]; and the third a population survey assessing long-term mortality among over 20,000 U.S. veterans who underwent either UPPP or CPAP therapy between October 1997 and September 2001 within the Veterans Affairs hospital system [118]. These results are summarized in Table 1. With the first study [117], subjective sleepiness, as measured with the Epworth Sleepiness scale (ESS), decreased to the same extent with RFTR & CPAP (−2.1 vs. −2.3, *p* = 0.005 & 0.02), as did the patients’ level of snoring, as measured with the SNORE25 (*p* < 0.001 vs. 0.005), both effects superior to sham RFTR (*p* < 0.001). However, objective sleepiness, measured as each patient’s average and shortest reaction time, only improved with RFTR (*p* = 0.03 and *p* = 0.02 versus sham therapy, respectively). In the non-randomized trial [83], RFTR and CPAP significantly reduced AHI versus baseline (28.5 → 15.7 vs. 29.6 → 16.1, both *p* < 0.001; no significant inter-treatment difference), with similar significant reductions noted for the ESS score (11.1 → 8.4, *p* = 0.003 vs. 10.8 → 8.2, *p* = 0.003; NS) and the percentage of time with oxygen saturation below 90 % (15.2 → 11.1 % vs. 14.3 → 10.7 %, both *p* < 0.001; NS). The two treatments also produced a similar increase in the patient’s lowest recorded nocturnal oxygen saturation level (88.4 → 93.5, *p* = 0.03 vs. 86.8 → 94.6 %, *p* < 0.001; NS). Overall, 53.8 and 52.4 % of patients were deemed to be treatment ‘responders’ (NS).

Perhaps most alarming of these results are those of the survival study [118], in which 1339 out of 18,754 patients on CPAP died over the course of observation (7.1 %) versus just 71 of 2072 (3.4 %) post-operatively. Adjusting for patient age, gender, race, year of treatment and co-morbid illnesses, there still was a 31 % (95 % CI: 3–67 %) increase in mortality among CPAP patients (*p* = 0.03) versus their post-UPPP counterparts. It may be that some patients receiving CPAP were considered too ill to be surgical candidates, a confounder that would falsely elevate the CPAP mortality rate. It is also important to acknowledge that the study authors were not able to adjust for OSA severity or account for CPAP adherence. Nonetheless, it is clear that all prior assumptions that CPAP is both more effective and safer than surgery as first-line treatment for OSA warrant re-evaluation.

In summary, though it is true that the evidence supporting CPAP over surgery as first-line therapy for OSA is more strongly supported by EBM level 1 evidence, closer inspection reveals major limitations of that evidence and reasons to suspect that this long-held assumption should now be questioned. Among these limitations are the very short-term follow-up of almost all CPAP trials (versus the longer follow-up of surgical trials); the very high degree of CPAP non-adherence that, in the vast majority of trials, was not accounted for by intent-to-treat analysis; failure to identify any significant advantage of CPAP over surgery in the two trials in which these two approaches were compared directly; and the apparent 30 % increased mortality observed in veterans who received CPAP versus surgical treatment in the U.S. between 1997 and 2001. Surgery appears to be clinically successful long-term in at least half of OSA patients, in terms of reducing their AHI to normal or near-normal levels. Given all of this, the time has come to rethink our CPAP-first approach to the OSA patient, especially in those patients in whom adherence, for whatever reason, might be considered an issue. Within that context however it is also imperative that surgeons understand that changing the current care ladder means also stressing the importance of correct patient selection and an appropriate consent process.

As a systematic review, this study is limited to the quality of the included studies. Because it is a collection of findings from various other studies, it provides an overview of the direction of literature but is unable to show new findings. This study is also limited in the fact that only English language articles are considered, which may introduce a language bias. However, studies are published from a variety of centers internationally. Because this study is not a meta-analysis, study results have not been statistically combined for more powerful results. One additional caveat is that studies were only graded by a traditional EBM approach as opposed to the more sophisticated Cochrane GRADE tool. This may introduce some level of bias to the priorities given to the various studies. However the large volume of literature reviewed for this paper should adequately compensate for that.

## Conclusion

This review illustrates the need for an in-depth, thorough and critical analysis of the available treatment options for the OSA patient. Although CPAP is often documented as the gold standard or mandatory first line therapy for patients with OSA, a careful assessment of the outcomes provided by the literature does not support this assertion, especially when the concept of CPAP adherence is taken into account. Clinicians should consider a patient-centered approach to care wherein the patient’s individual anatomical characteristics are evaluated in context of their OSA severity and treatment goals, and then tailor intervention to individual needs. In many patients beneficial surgical results may supplant the role of the CPAP machine when considering first line therapy.

## Additional files

Additional file 1: Table S1. CPAP comparisons. (DOCX 60 kb) Additional file 2: Table S2. Surgery findings. (DOCX 43 kb)

## References

[1] Giles TL, Lasserson TJ, Smith B, White J, Wright JJ, Cates CJ. Continuous positive airways pressure for obstructive sleep apnoea in adults: a Cochrane Collaboration Review. Etobicoke, Canada: John Wiley & Sons, Ltd; 2008. Issue 4, 1–103.

[2] McDaid C, Griffin S, Weatherly H, et al. Continuous positive airway pressure devices for the treatment of obstructive sleep apnoea-hypopnoea syndrome: a systematic review and economic analysis. Southampton, UK: National Institutes of Health Research Health Technology Assessment Program; 2009.1–162. 10.3310/hta13040 19103134

[3] Sundaram S, Lim J, Lasserson TJ. Surgery for obstructive sleep apnoea in adults: A Cochrane Collaboration Review. Etobicoke, Canada: John Wiley & Sons, Ltd; 2009. 1, 1–72.

[4] Aarab G, Lobbezoo F, Heymans MW, Hamburger HL, Naeije M (2011). Long-term follow-up of a randomized controlled trial of oral appliance therapy in obstructive sleep apnea. Respiration.

[5] Aarab G, Lobbezoo F, Hamburger HL, Naeije M (2011). Oral appliance therapy versus nasal continuous positive airway pressure in obstructive sleep apnea: a randomized, placebo-controlled trial. Respiration.

[6] Ancoli-Israel S, Palmer BW, Cooke JR (2008). Cognitive effects of treating obstructive sleep apnea in Alzheimer’s disease: a randomized controlled study. J Am Geriatr Soc.

[7] Arias MA, Garcia-Rio F, Alonso-Fernandez A, Martinez I, Villamor J (2006). Pulmonary hypertension in obstructive sleep apnoea: effects of continuous positive airway pressure: a randomized, controlled cross-over study. Eur Heart J.

[8] Bakker J, Campbell A, Neill A (2010). Randomized controlled trial comparing flexible and continuous positive airway pressure delivery: effects on adherence, objective and subjective sleepiness and vigilance. Sleep.

[9] Barbe F, Mayoralas LR, Duran J (2001). Treatment with continuous positive airway pressure is not effective in patients with sleep apnea but no daytime sleepiness. a randomized, controlled trial. Ann Intern Med.

[10] Barbe F, Duran-Cantolla J, Capote F (2010). Long-term effect of continuous positive airway pressure in hypertensive patients with sleep apnea. Am J Respir Crit Care Med.

[11] Becker HF, Jerrentrup A, Ploch T (2003). Effect of nasal continuous positive airway pressure treatment on blood pressure in patients with obstructive sleep apnea. Circulation.

[12] Campos-Rodriguez F, Grilo-Reina A, Perez-Ronchel J (2006). Effect of continuous positive airway pressure on ambulatory BP in patients with sleep apnea and hypertension: a placebo-controlled trial. Chest.

[13] Coughlin SR, Mawdsley L, Mugarza JA, Wilding P, Calverley PM (2007). Cardiovascular and metabolic effects of CPAP in obese males with OSA. Eur Respir J.

[14] Cross MD, Mills NL, Al-Abri M (2008). Continuous positive airway pressure improves vascular function in obstructive sleep apnoea/hypopnoea syndrome: a randomised controlled trial. Thorax.

[15] Dimsdale JE, Loredo JS, Profant J (2000). Effect of continuous positive airway pressure on blood pressure : a placebo trial. Hypertension.

[16] Duran-Cantolla J, Aizpuru F, Montserrat JM (2010). Continuous positive airway pressure as treatment for systemic hypertension in people with obstructive sleep apnoea: randomised controlled trial. BMJ.

[17] Egea CJ, Aizpuru F, Pinto JA (2008). Cardiac function after CPAP therapy in patients with chronic heart failure and sleep apnea: a multicenter study. Sleep Med.

[18] Henke KG, Grady JJ, Kuna ST (2001). Effect of nasal continuous positive airway pressure on neuropsychological function in sleep apnea-hypopnea syndrome. A randomized, placebo-controlled trial. Am J Respir Crit Care Med.

[19] Hui DS, To KW, Ko FW (2006). Nasal CPAP reduces systemic blood pressure in patients with obstructive sleep apnoea and mild sleepiness. Thorax.

[20] Jenkinson C, Davies RJ, Mullins R, Stradling JR (1999). Comparison of therapeutic and subtherapeutic nasal continuous positive airway pressure for obstructive sleep apnoea: a randomised prospective parallel trial. Lancet.

[21] Jenkinson C, Davies RJ, Mullins R, Stradling JR (2001). Long-term benefits in self-reported health status of nasal continuous positive airway pressure therapy for obstructive sleep apnoea. QJM.

[22] Lojander J, Maasilta P, Partinen M, Brander PE, Salmi T, Lehtonen H (1996). Nasal-CPAP, surgery, and conservative management for treatment of obstructive sleep apnea syndrome. Chest.

[23] Loredo JS, Ancoli-Israel S, Kim EJ, Lim WJ, Dimsdale JE (2006). Effect of continuous positive airway pressure versus supplemental oxygen on sleep quality in obstructive sleep apnea: a placebo-CPAP-controlled study. Sleep.

[24] Marshall NS, Neill AM, Campbell AJ, Sheppard DS (2005). Randomised controlled crossover trial of humidified continuous positive airway pressure in mild obstructive sleep apnoea. Thorax.

[25] Marshall NS, Neill AM, Campbell AJ (2008). Randomised trial of adherence with flexible (C-Flex) and standard continuous positive airway pressure for severe obstructive sleep apnea. Sleep Breath.

[26] Montserrat JM, Ferrer M, Hernandez L (2001). Effectiveness of CPAP treatment in daytime function in sleep apnea syndrome: a randomized controlled study with an optimized placebo. Am J Respir Crit Care Med.

[27] Norman D, Loredo JS, Nelesen RA (2006). Effects of continuous positive airway pressure versus supplemental oxygen on 24-hour ambulatory blood pressure. Hypertension.

[28] Pepperell JC, Ramdassingh-Dow S, Crosthwaite N (2002). Ambulatory blood pressure after therapeutic and subtherapeutic nasal continuous positive airway pressure for obstructive sleep apnoea: a randomised parallel trial. Lancet.

[29] Robinson GV, Smith DM, Langford BA, Davies RJ, Stradling JR (2006). Continuous positive airway pressure does not reduce blood pressure in nonsleepy hypertensive OSA patients. Eur Respir J.

[30] Sharma SK, Agrawal S, Damodaran D (2011). CPAP for the metabolic syndrome in patients with obstructive sleep apnea. N Engl J Med.

[31] Siccoli MM, Pepperell JC, Kohler M, Craig SE, Davies RJ, Stradling JR (2008). Effects of continuous positive airway pressure on quality of life in patients with moderate to severe obstructive sleep apnea: data from a randomized controlled trial. Sleep.

[32] West SD, Nicoll DJ, Wallace TM, Matthews DR, Stradling JR (2007). Effect of CPAP on insulin resistance and HbA1c in men with obstructive sleep apnoea and type 2 diabetes. Thorax.

[33] Engleman HM, Martin SE, Deary IJ, Douglas NJ (1994). Effect of continuous positive airway pressure treatment on daytime function in sleep apnoea/hypopnoea syndrome. Lancet.

[34] Spicuzza L, Bernardi L, Balsamo R, Ciancio N, Polosa R, Di MG (2006). Effect of treatment with nasal continuous positive airway pressure on ventilatory response to hypoxia and hypercapnia in patients with sleep apnea syndrome. Chest.

[35] Barnes M, Houston D, Worsnop CJ (2002). A randomized controlled trial of continuous positive airway pressure in mild obstructive sleep apnea. Am J Respir Crit Care Med.

[36] Barnes M, McEvoy RD, Banks S (2004). Efficacy of positive airway pressure and oral appliance in mild to moderate obstructive sleep apnea. Am J Respir Crit Care Med.

[37] Engleman HM, Gough K, Martin SE, Kingshott RN, Padfield PL, Douglas NJ (1996). Ambulatory blood pressure on and off continuous positive airway pressure therapy for the sleep apnea/hypopnea syndrome: effects in “non-dippers”. Sleep.

[38] Engleman HM, Martin SE, Deary IJ, Douglas NJ (1997). Effect of CPAP therapy on daytime function in patients with mild sleep apnoea/hypopnoea syndrome. Thorax.

[39] Engleman HM, Martin SE, Kingshott RN, Mackay TW, Deary IJ, Douglas NJ (1998). Randomised placebo controlled trial of daytime function after continuous positive airway pressure (CPAP) therapy for the sleep apnoea/hypopnoea syndrome. Thorax.

[40] Engleman HM, Kingshott RN, Wraith PK, Mackay TW, Deary IJ, Douglas NJ (1999). Randomized placebo-controlled crossover trial of continuous positive airway pressure for mild sleep Apnea/Hypopnea syndrome. Am J Respir Crit Care Med.

[41] Faccenda JF, Mackay TW, Boon NA, Douglas NJ (2001). Randomized placebo-controlled trial of continuous positive airway pressure on blood pressure in the sleep apnea-hypopnea syndrome. Am J Respir Crit Care Med.

[42] McArdle N, Douglas NJ (2001). Effect of continuous positive airway pressure on sleep architecture in the sleep apnea-hypopnea syndrome: a randomized controlled trial. Am J Respir Crit Care Med.

[43] McArdle N, Kingshott R, Engleman HM, Mackay TW, Douglas NJ (2001). Partners of patients with sleep apnoea/hypopnoea syndrome: effect of CPAP treatment on sleep quality and quality of life. Thorax.

[44] Ballester E, Badia JR, Hernandez L (1999). Evidence of the effectiveness of continuous positive airway pressure in the treatment of sleep apnea/hypopnea syndrome. Am J Respir Crit Care Med.

[45] Barbe F, Duran-Cantolla J, Sanchez-de-la-Torre M (2012). Effect of continuous positive airway pressure on the incidence of hypertension and cardiovascular events in nonsleepy patients with obstructive sleep apnea: a randomized controlled trial. JAMA.

[46] Chakravorty I, Cayton RM, Szczepura A (2002). Health utilities in evaluating intervention in the sleep apnoea/hypopnoea syndrome. Eur Respir J.

[47] Drager LF, Bortolotto LA, Figueiredo AC, Krieger EM, Lorenzi GF (2007). Effects of continuous positive airway pressure on early signs of atherosclerosis in obstructive sleep apnea. Am J Respir Crit Care Med.

[48] Drager LF, Pedrosa RP, Diniz PM (2011). The effects of continuous positive airway pressure on prehypertension and masked hypertension in men with severe obstructive sleep apnea. Hypertension.

[49] Hsu CY, Vennelle M, Li HY, Engleman HM, Dennis MS, Douglas NJ (2006). Sleep-disordered breathing after stroke: a randomised controlled trial of continuous positive airway pressure. J Neurol Neurosurg Psychiatry.

[50] Kaneko Y, Floras JS, Usui K (2003). Cardiovascular effects of continuous positive airway pressure in patients with heart failure and obstructive sleep apnea. N Engl J Med.

[51] Lam B, Sam K, Mok WY (2007). Randomised study of three non-surgical treatments in mild to moderate obstructive sleep apnoea. Thorax.

[52] Mansfield DR, Gollogly NC, Kaye DM, Richardson M, Bergin P, Naughton NT (2004). Controlled trial of continuous positive airway pressure in obstructive sleep apnea and heart failure. Am J Respir Crit Care Med.

[53] Monasterio C, Vidal S, Duran J (2001). Effectiveness of continuous positive airway pressure in mild sleep apnea-hypopnea syndrome. Am J Respir Crit Care Med.

[54] Redline S, Adams N, Strauss ME, Roebuck T, Winters M, Rosenberg C (1998). Improvement of mild sleep-disordered breathing with CPAP compared with conservative therapy. Am J Respir Crit Care Med.

[55] Engleman HM, McDonald JP, Graham D (2002). Randomized crossover trial of two treatments for sleep apnea/hypopnea syndrome: continuous positive airway pressure and mandibular repositioning splint. Am J Respir Crit Care Med.

[56] Ferguson KA, Ono T, Lowe AA, Keenan SP, Fleetham JA (1996). A randomized crossover study of an oral appliance vs nasal-continuous positive airway pressure in the treatment of mild-moderate obstructive sleep apnea. Chest.

[57] Ferguson KA, Ono T, Lowe AA, al-Majed S, Love LL, Fleetham JA (1997). A short-term controlled trial of an adjustable oral appliance for the treatment of mild to moderate obstructive sleep apnoea. Thorax.

[58] Gagnadoux F, Fleury B, Vielle B (2009). Titrated mandibular advancement versus positive airway pressure for sleep apnoea. Eur Respir J.

[59] Hoekema A, Stegenga B, Wijkstra PJ, van der Hoeven JH, Meinesz AF, De Bont LG (2008). Obstructive sleep apnea therapy. J Dent Res.

[60] Randerath WJ, Heise M, Hinz R, Ruehle KH (2002). An individually adjustable oral appliance vs continuous positive airway pressure in mild-to-moderate obstructive sleep apnea syndrome. Chest.

[61] Tan YK, L’Estrange PR, Luo YM (2002). Mandibular advancement splints and continuous positive airway pressure in patients with obstructive sleep apnoea: a randomized cross-over trial. Eur J Orthod.

[62] Trzepizur W, Gagnadoux F, Abraham P (2009). Microvascular endothelial function in obstructive sleep apnea: Impact of continuous positive airway pressure and mandibular advancement. Sleep Med.

[63] Doff MH, Hoekema A, Wijkstra PJ (2013). Oral appliance versus continuous positive airway pressure in obstructive sleep apnea syndrome: a 2-year follow-up. Sleep.

[64] Jokic R, Klimaszewski A, Crossley M, Sridhar G, Fitzpatrick MF (1999). Positional treatment vs continuous positive airway pressure in patients with positional obstructive sleep apnea syndrome. Chest.

[65] Permut I, Diaz-Abad M, Chatila W (2010). Comparison of positional therapy to CPAP in patients with positional obstructive sleep apnea. J Clin Sleep Med.

[66] Skinner MA, Kingshott RN, Jones DR, Homan SD, Taylor DR (2004). Elevated posture for the management of obstructive sleep apnea. Sleep Breath.

[67] Skinner MA, Kingshott RN, Jones DR, Taylor DR (2004). Lack of efficacy for a cervicomandibular support collar in the management of obstructive sleep apnea. Chest.

[68] Ballard RD, Gay PC, Strollo PJ (2007). Interventions to improve adherence in sleep apnea patients previously non-compliant with continuous positive airway pressure. J Clin Sleep Med.

[69] Engleman HM, Martin SE, Douglas NJ (1994). Adherence with CPAP therapy in patients with the sleep apnoea/hypopnoea syndrome. Thorax.

[70] Kohler M, Stoewhas AC, Ayers L (2011). Effects of continuous positive airway pressure therapy withdrawal in patients with obstructive sleep apnea: a randomized controlled trial. Am J Respir Crit Care Med.

[71] Kryger MH, Berry RB, Massie CA (2011). Long-term use of a nasal expiratory positive airway pressure (EPAP) device as a treatment for obstructive sleep apnea (OSA). J Clin Sleep Med.

[72] Kushida CA, Berry RB, Blau A (2011). Positive airway pressure initiation: a randomized controlled trial to assess the impact of therapy mode and titration process on efficacy, adherence, and outcomes. Sleep.

[73] Roecklein KA, Schumacher JA, Gabriele JM, Fagan C, Baran AS, Richert AC (2010). Personalized feedback to improve CPAP adherence in obstructive sleep apnea. Behav Sleep Med.

[74] Ruhle KH, Franke KJ, Domanski U, Nilius G (2010). Quality of life, adherence, sleep and nasopharyngeal side effects during CPAP therapy with and without controlled heated humidification. Sleep Breath.

[75] Ryan S, Doherty LS, Nolan GM, McNicholas WT (2009). Effects of heated humidification and topical steroids on adherence, nasal symptoms, and quality of life in patients with obstructive sleep apnea syndrome using nasal continuous positive airway pressure. J Clin Sleep Med.

[76] To KW, Chan WC, Choo KL, Lam WK, Wong KK, Hui DS (2008). A randomized cross-over study of auto-continuous positive airway pressure versus fixed-continuous positive airway pressure in patients with obstructive sleep apnoea. Respirology.

[77] Vennelle M, White S, Riha RL, Mackay TW, Engleman HM, Douglas NJ (2010). Randomized controlled trial of variable-pressure versus fixed-pressure continuous positive airway pressure (CPAP) treatment for patients with obstructive sleep apnea/hypopnea syndrome (OSAHS). Sleep.

[78] Weaver TE, Maislin G, Dinges DF (2007). Relationship between hours of CPAP use and achieving normal levels of sleepiness and daily functioning. Sleep.

[79] Wolkove N, Baltzan M, Kamel H, Dabrusin R, Palayew M (2008). Long-term adherence with continuous positive airway pressure in patients with obstructive sleep apnea. Can Respir J.

[80] Cahali MB (2003). Lateral pharyngoplasty: a new treatment for obstructive sleep apnea hypopnea syndrome. Laryngoscope.

[81] Friedman M, Ibraham H, Lee G, Joseph NJ (2003). Combined uvulopalatopharyngoplasty and radiofrequency tongue base reduction for treatment of obstructive sleep apnea/hypopnea syndrome. Otolaryngol Head Neck Surg.

[82] Hendler BH, Costello BJ, Silverstein K, Yen D, Goldberg A (2001). A protocol for uvulopalatopharyngoplasty, mortised genioplasty, and maxillomandibular advancement in patients with obstructive sleep apnea: an analysis of 40 cases. J Oral Maxillofac Surg.

[83] Lee NR, Givens CD, Wilson J, Robins RB (1999). Staged surgical treatment of obstructive sleep apnea syndrome: a review of 35 patients. J Oral Maxillofac Surg.

[84] Li KK (2005). Surgical therapy for adult obstructive sleep apnea. Sleep Med Rev.

[85] Miller FR, Watson D, Malis D (2002). Role of the tongue base suspension suture with The Repose System bone screw in the multilevel surgical management of obstructive sleep apnea. Otolaryngol Head Neck Surg.

[86] Nelson LM (2001). Combined temperature-controlled radiofrequency tongue reduction and UPPP in apnea surgery. Ear Nose Throat J.

[87] Neruntarat C (2003). Hyoid myotomy with suspension under local anesthesia for obstructive sleep apnea syndrome. Eur Arch Otorhinolaryngol.

[88] Neruntarat C (2003). Genioglossus advancement and hyoid myotomy under local anesthesia. Otolaryngol Head Neck Surg.

[89] Neruntarat C (2011). Uvulopalatal flap for obstructive sleep apnea: short-term and long-term results. Laryngoscope.

[90] Terris DJ, Kunda LD, Gonella MC (2002). Minimally invasive tongue base surgery for obstructive sleep apnoea. J Laryngol Otol.

[91] Vilaseca I, Morello A, Montserrat JM, Santamaria J, Iranzo A (2002). Usefulness of uvulopalatopharyngoplasty with genioglossus and hyoid advancement in the treatment of obstructive sleep apnea. Arch Otolaryngol Head Neck Surg.

[92] Walker-Engstrom ML, Wilhelmsson B, Tegelberg A, Dimenas E, Ringqvist I (2000). Quality of life assessment of treatment with dental appliance or UPPP in patients with mild to moderate obstructive sleep apnoea. A prospective randomized 1-year follow-up study. J Sleep Res.

[93] Walker-Engstrom ML, Tegelberg A, Wilhelmsson B, Ringqvist I (2002). 4-year follow-up of treatment with dental appliance or uvulopalatopharyngoplasty in patients with obstructive sleep apnea: a randomized study. Chest.

[94] Weaver EM, Woodson BT, Yueh B (2011). Studying Life Effects & Effectiveness of Palatopharyngoplasty (SLEEP) Study: subjective outcomes of isolated uvulopalatopharyngoplasty. Otolaryngol Head Neck Surg.

[95] Wilhelmsson B, Tegelberg A, Walker-Engstrom ML (1999). A prospective randomized study of a dental appliance compared with uvulopalatopharyngoplasty in the treatment of obstructive sleep apnoea. Acta Otolaryngol.

[96] Aneeza WH, Marina MB, Razif MY, Azimatun NA, Asma A, Sani A (2011). Effects of uvulopalatopharyngoplasty: a seven year review. Med J Malaysia.

[97] Baradaranfar MH, Edalatkhah M, Dadgarnia MH (2015). The effect of uvulopalatopharyngoplasty with tonsillectomy in patients with obstructive sleep apnea. Indian J Otolaryngol Head Neck Surg.

[98] Lee JM, Weinstein GS, O’Malley BW, Thaler ER (2012). Transoral robot-assisted lingual tonsillectomy and uvulopalatopharyngoplasty for obstructive sleep apnea. Ann Otol Rhinol Laryngol.

[99] MacKay SG, Carney AS, Woods C (2013). Modified uvulopalatopharyngoplasty and coblation channeling of the tongue for obstructive sleep apnea: a multi-centre Australian trial. J Clin Sleep Med.

[100] Pang KP, Tan R, Puraviappan P, Terris DJ (2009). Anterior palatoplasty for the treatment of OSA: three-year results. Otolaryngol Head Neck Surg.

[101] Verse T, Baisch A, Maurer JT, Stuck BA, Hormann K (2006). Multilevel surgery for obstructive sleep apnea: short-term results. Otolaryngol Head Neck Surg.

[102] Weaver EM, Maynard C, Yueh B (2004). Survival of veterans with sleep apnea: continuous positive airway pressure versus surgery. Otolaryngol Head Neck Surg.

[103] Yang HB, Wang Y, Dong MM (2012). Effect of Han-uvulopalatopharyngoplasty on flow-mediated dilatation in patients with moderate or severe obstructive sleep apnea syndrome. Acta Otolaryngol.

[104] Yaremchuk K, Tacia B, Peterson E, Roth T (2011). Change in Epworth Sleepiness Scale after surgical treatment of obstructive sleep apnea. Laryngoscope.

[105] Yu S, Liu F, Wang Q (2010). Effect of revised UPPP surgery on ambulatory BP in sleep apnea patients with hypertension and oropharyngeal obstruction. Clin Exp Hypertens.

[106] Djupesland G, Schrader H, Lyberg T, Refsum H, Lilleas F, Godtlibsen OB (1992). Palatopharyngoglossoplasty in the treatment of patients with obstructive sleep apnea syndrome. Acta Otolaryngol Suppl.

[107] Ramirez SG, Loube DI (1996). Inferior sagittal osteotomy with hyoid bone suspension for obese patients with sleep apnea. Arch Otolaryngol Head Neck Surg.

[108] Elasfour A, Miyazaki S, Itasaka Y, Yamakawa K, Ishikawa K, Togawa K (1998). Evaluation of uvulopalatopharyngoplasty in treatment of obstructive sleep apnea syndrome. Acta Otolaryngol Suppl.

[109] Hendler B, Silverstein K, Giannakopoulos H, Costello BJ (2001). Mortised genioplasty in the treatment of obstructive sleep apnea: an historical perspective and modification of design. Sleep Breath.

[110] Dattilo DJ, Drooger SA (2004). Outcome assessment of patients undergoing maxillofacial procedures for the treatment of sleep apnea: comparison of subjective and objective results. J Oral Maxillofac Surg.

[111] Dattilo DJ, Aynechi M (2007). Modification of the anterior mandibular osteotomy for genioglossus advancement with hyoid suspension for obstructive sleep apnea. J Oral Maxillofac Surg.

[112] Li HY, Wang PC, Hsu CY, Chen NH, Lee LA, Fang TJ (2004). Same-stage palatopharyngeal and hypopharyngeal surgery for severe obstructive sleep apnea. Acta Otolaryngol.

[113] Hathaway B, Johnson JT (2006). Safety of uvulopalatopharyngoplasty as outpatient surgery. Otolaryngol Head Neck Surg.

[114] Baugh R, Burke B, Fink B, Garcia R, Kominsky A, Yaremchuk K (2013). Safety of outpatient surgery for obstructive sleep apnea. Otolaryngol Head Neck Surg.

[115] Rotenberg BW, Theriault J, Gottesman S (2014). Redefining the timing of surgery for obstructive sleep apnea in anatomically favorable patients. Laryngoscope.

[116] Cahali MB, Formigoni GG, Gebrim EM, Miziara ID (2004). Lateral pharyngoplasty versus uvulopalatopharyngoplasty: a clinical, polysomnographic and computed tomography measurement comparison. Sleep.

[117] Thomas AJ, Chavoya M, Terris DJ (2003). Preliminary findings from a prospective, randomized trial of two tongue-base surgeries for sleep-disordered breathing. Otolaryngol Head Neck Surg.

[118] Bettega G, Pepin JL, Veale D, Deschaux C, Raphael B, Levy P (2000). Obstructive sleep apnea syndrome. fifty-one consecutive patients treated by maxillofacial surgery. Am J Respir Crit Care Med.

[119] Li KK, Powell NB, Riley RW, Troell RJ, Guilleminault C (2000). Long-term results of maxillomandibular advancement surgery. Sleep Breath.

[120] Ferguson KA, Heighway K, Ruby RR (2003). A randomized trial of laser-assisted uvulopalatoplasty in the treatment of mild obstructive sleep apnea. Am J Respir Crit Care Med.

[121] Finkelstein Y, Stein G, Ophir D, Berger R, Berger G (2002). Laser-assisted uvulopalatoplasty for the management of obstructive sleep apnea: myths and facts. Arch Otolaryngol Head Neck Surg.

[122] Kyrmizakis DE, Chimona TS, Papadakis CE (2003). Laser-assisted uvulopalatoplasty for the treatment of snoring and mild obstructive sleep apnea syndrome. J Otolaryngol.

[123] Lin CC, Lee KS, Chang KC, Wu KM, Chou CS (2006). Effect of laser-assisted uvulopalatoplasty on oral airway resistance during wakefulness in obstructive sleep apnea syndrome. Eur Arch Otorhinolaryngol.

[124] Mickelson SA (1996). Laser-assisted uvulopalatoplasty for obstructive sleep apnea. Laryngoscope.

[125] Atef A, Mosleh M, Hesham M, Fathi A, Hassan M, Fawzy M (2005). Radiofrequency vs laser in the management of mild to moderate obstructive sleep apnoea: does the number of treatment sessions matter?. J Laryngol Otol.

[126] Ceylan K, Emir H, Kizilkaya Z (2009). First-choice treatment in mild to moderate obstructive sleep apnea: single-stage, multilevel, temperature-controlled radiofrequency tissue volume reduction or nasal continuous positive airway pressure. Arch Otolaryngol Head Neck Surg.

[127] Powell NB, Riley RW, Guilleminault C (1999). Radiofrequency tongue base reduction in sleep-disordered breathing: A pilot study. Otolaryngol Head Neck Surg.

[128] Riley RW, Powell NB, Li KK, Weaver EM, Guilleminault C (2003). An adjunctive method of radiofrequency volumetric tissue reduction of the tongue for OSAS. Otolaryngol Head Neck Surg.

[129] Steward DL, Weaver EM, Woodson BT (2005). Multilevel temperature-controlled radiofrequency for obstructive sleep apnea: extended follow-up. Otolaryngol Head Neck Surg.

[130] Stuck BA, Starzak K, Hein G, Verse T, Hormann K, Maurer JT (2004). Combined radiofrequency surgery of the tongue base and soft palate in obstructive sleep apnoea. Acta Otolaryngol.

[131] Woodson BT, Nelson LM, Mickelson S, Huntley T, Sher A (2001). A multi-institutional study of radiofrequency volumetric tissue reduction for OSAS. Otolaryngol Head Neck Surg.

[132] Woodson BT (2001). A tongue suspension suture for obstructive sleep apnea and snorers. Otolaryngol Head Neck Surg.

[133] Woodson BT, Steward DL, Weaver EM, Javaheri S (2003). A randomized trial of temperature-controlled radiofrequency, continuous positive airway pressure, and placebo for obstructive sleep apnea syndrome. Otolaryngol Head Neck Surg.

[134] Bassiouny A, El Salamawy A, Abd El-Tawab M, Atef A (2007). Bipolar radiofrequency treatment for snoring with mild to moderate sleep apnea: a comparative study between the radiofrequency assisted uvulopalatoplasty technique and the channeling technique. Eur Arch Otorhinolaryngol.

[135] Friedman M, Schalch P, Lin HC, Kakodkar KA, Joseph NJ, Mazloom N (2008). Palatal implants for the treatment of snoring and obstructive sleep apnea/hypopnea syndrome. Otolaryngol Head Neck Surg.

[136] Nordgard S, Stene BK, Skjostad KW (2006). Soft palate implants for the treatment of mild to moderate obstructive sleep apnea. Otolaryngol Head Neck Surg.

[137] Pang KP, Terris DJ (2007). Modified cautery-assisted palatal stiffening operation: new method for treating snoring and mild obstructive sleep apnea. Otolaryngol Head Neck Surg.

[138] Walker RP, Levine HL, Hopp ML, Greene D, Pang K (2006). Palatal implants: a new approach for the treatment of obstructive sleep apnea. Otolaryngol Head Neck Surg.

[139] Walker RP, Levine HL, Hopp ML, Greene D (2007). Extended follow-up of palatal implants for OSA treatment. Otolaryngol Head Neck Surg.

[140] Back LJ, Liukko T, Rantanen I (2009). Radiofrequency surgery of the soft palate in the treatment of mild obstructive sleep apnea is not effective as a single-stage procedure: A randomized single-blinded placebo-controlled trial. Laryngoscope.

[141] Huang TW, Cheng PW, Fang KM (2011). Concurrent palatal implants and uvulopalatal flap: safe and effective office-based procedure for selected patients with snoring and obstructive sleep apnea syndrome. Laryngoscope.

[142] Kezirian EJ, Weaver EM, Yueh B (2004). Incidence of serious complications after uvulopalatopharyngoplasty. Laryngoscope.

[143] Strocker AM, Cohen AN, Wang MB (2008). The safety of outpatient UPPP for obstructive sleep apnea: a retrospective review of 40 cases. Ear Nose Throat J.

[144] Rotenberg B, Hu A, Fuller J, Bureau Y, Arra I, Sen M (2010). The early postoperative course of surgical sleep apnea patients. Laryngoscope.

[145] Pang KP, Siow JK, Tseng P (2012). Safety of multilevel surgery in obstructive sleep apnea: a review of 487 cases. Arch Otolaryngol Head Neck Surg.

[146] Kandasamy T, Wright ED, Fuller J, Rotenberg BW (2013). The incidence of early post-operative complications following uvulopalatopharyngoplasty: identification of predictive risk factors. J Otolaryngol Head Neck Surg.

