# Supplementary material for: Reconsidering first-line treatment for obstructive sleep apnea: a systematic review of the literature
Source: J Otolaryngol Head Neck Surg. 2016 Apr 6;45:23. doi: 10.1186/s40463-016-0136-4 (PMC4822285; doi:10.1186/s40463-016-0136-4)
Supplement: Additional file 1: Table S1. — CPAP comparisons. (DOCX 60 kb) [file 40463_2016_136_MOESM1_ESM.docx]

Table S1: CPAP comparisons

| First author | EBM | Study | | Length of | | Study | Study |
| --- | --- | --- | --- | --- | --- | --- | --- |
| year | rating | design | | FU | | findings | limitations/issues |
| **CPAP vs. sham CPAP** | |  | |  | |  |  |
| Jenkinson | 1 | RCT | | 4 wks | | Vs. sham, nCPAP → ↓ESS (15.5→7.0 vs. 15.0→13.0, p < 0.001) | Short FU, though non-randomly selected cohort followed |
| 1999 |  |  | |  | |  | on CPAP for a further 2 years (Jenkinson, 2001) |
|  |  |  | |  | |  |  |
| Dimsdale | 1 | RCT | | 1 wks | | RDI ↓53.6 → 3.2 on CPAP, 41.7 → 28.1 on sham (p = 0.001) | Short FU; CPAP group had ↑ BL μsBP and dBP (128/82 vs. 123/78) |
| 2000 |  |  | |  | | NT μaBP ↓ greater on CPAP (5 vs. 1 mmHg, p = 0.03) | constant contact w/ research team ↑ compliance |
|  |  |  | |  | |  |  |
| Barbé | 1 | RCT | | 6 wks | | No change in ESS, QoL, cognitive fxn or arterial BP | No objective measure of OSA (e.g., AHI) as an outcome; |
| 2001 |  |  | |  | |  | many variables normal at baseline (e.g., no DT sleepiness) |
|  |  |  | |  | |  |  |
| Henke | 1 | XO | | 5/3** wk | | In both groups on CPAP, AHI, AI and # desat. ↓ & LSAT | Bizarre study design; analysis difficult |
| 2001 |  |  | |  | | no difference in changes in neuropsych. fxn on CPAP vs. sham CPAP | (groups on different stages of Rx for different lengths of time) |
|  |  |  | |  | |  |  |
| Montserrat | 1 | RCT + | | 6 wks | | Vs sham, CPAP → ↓ESS & total SASH symptoms, ↑ FOSQ vigilance after RCT | Short FU; 2nd period of Rx (sham pts on therapeutic CPAP x 6 wk) |
| 2001 |  | cohort | |  | |  | was unblind |
|  |  |  | |  | |  |  |
| Pepperell | 1 | RCT | | 4 wks | | nCPAP, ↓24-hour μBP (p = 0.001), μBP during sleep (p = 0.03) | High drop-out rate; no measure of OSA severity or symtoms on FU |
| 2002 |  |  | |  | |  |  |
|  |  |  | |  | |  |  |
| Becker | 2 | RCT | | 9 wks | | CPAP ↓ESS, AHI & MAP (9.9mmHg), ↑SaO2 vs. non-therapeutic CPAP | Very high dropout rate; greater CPAP compliance likely due |
| 2003 |  |  | |  | |  | to this being an in-hospital study |
|  |  |  | |  | |  |  |
| Arias | 1 | XO | | 12 wks | | nCPAP → ↓mitral deceleration (p < 0.01) and isovolumic relaxation (p < 0.05) | Small sample; no follow-up of OSA severity (e.g., AHI, ESS) |
| 2005 |  |  | |  | |  |  |
|  |  |  | |  | |  |  |
| Marshall | 1 | XO | | 3 wks | | Vs sham, CPAP → ↓ESS (p = 0.04) & FOSQ vigilance score (all p < 0.05); | Small sample; short FU; no statistical adjustment for multiple comparisons |
| 2005 |  |  | |  | |  | despite 22 outcomes of interest (CPAP affected 4/22) |
|  |  |  | |  | |  |  |
| Arias | 2 | XO | | 12 wks | | Pulmonary artery pressure ↓ w/ CPAP (28.8 vs. 24.0, p < 0.0001) | Small heterogenious sample (10 w/ pulmonary HTN; 13 w/o); |
| 2006 |  |  | |  | |  | no follow-up of OSA severity (e.g., AHI, ESS) |
|  |  |  | |  | |  |  |
| Campos | 1 | RCT | | 4 wks | | ESS ↓ in therapeutic but not subtherapeutic CPAP group (P<0.05); | No inter-group statistical comparisons (vs. baseline only); |
| 2006 |  |  | |  | | no ↓ in BP | no follow-up AHI |
|  |  |  | |  | |  |  |
| Doff | 1 | RCT | | 4 wks | | Vs sham, nCPAP → ↓ AHI (55.3 → 2.1 vs. 59.2 → 57.0; p < 0.001) | Small study; short FU; no measure of OSA symptoms; strict exclusion criteria |
| 2006 |  |  | |  | |  | (e.g., hypertension) limit generalizability of findings |
|  |  |  | |  | |  |  |
| Hui | 1 | RCT | | 12 wks | | Vs sham nCPAP, nCPAP → ↓ 24-hour dBP (↓2.4 vs. ↑1.1), | 44/100 patients initially identified for study refused to participate; |
| 2006 |  |  | |  | | ↓ sleep-time sBP (↓4.1 vs. ↑2.2) | tended to have worse OSA & higher BP (the group at greatest need) |
|  |  |  | |  | |  |  |
| Loredo | 1 | RCT | | 2 wks | | CPAP → ↓ AHI, TAI & stage 1 sleep & ↑ mean O2sat & REM sleep | Few subjects per group; very short FU; high drop-out rate |
| 2006 |  |  | |  | |  |  |
|  |  |  | |  | |  |  |
| Norman | 1 | RCT | | 2 wks | | CPAP → ↓ AHI & ODI, NT sBP, dBP & MAP, & DT dBP | Short FU; CPAP group older, heavier, & had worse OSA, higher sBP, dBP & MAP |
| 2006 |  |  | |  | | and MAP & ↑ NT MSAT & LSAT vs. sham; | than other groups (only sBP achieving statistical significance) |
|  |  |  | |  | |  |  |
| Robinson | 1 | XO | | 4 wks | | No intergroup difference in ↓ 24-hr BP (all p > 0.30) | Heterogenous population re: cause of HTN & anti-HTN medications used; |
| 2006 |  |  | |  | |  | short FU (4 weeks) |
|  |  |  | |  | |  |  |
| Coughlin | 1 | XO | | 6 wks | | ESS, sBP, dBP and MAP ↓ on CPAP vs. sham (p < 0.01) | No follow-up AHI; follow-up of only 6 weeks |
| 2007 |  |  | |  | |  |  |
|  |  |  | |  | |  |  |
| West | 1 | RCT | | 12 wks | | nCPAP → ↓ ESS (p = 0.01) & ↑ MWT (p = 0.001) & SAQLI score (p = 0.04). | Small study; faulty equipment, randomization problems; |
| 2007 |  |  | |  | | No effect of either Rx on glycaemic control or insulin resistance | no objective OSA severity measure (e.g. AHI) as outcome |
|  |  |  | |  | |  |  |
| Ancoli-Israel | 2 | RCT | | 6/3 wks | | AHI ↓ from 29.7 → 6.4 on CPAP vs. 26.9 → 34.6 on sham (p < 0.001) | 25% dropout rate; resulting small sample disallowed inter-Rx comparisons |
| 2008 |  |  | |  | |  | of cognitive & neuropsych scores |
|  |  |  | |  | |  |  |
| Cross | 1 | XO | | 6 wks | | no differnce in heart rate, sBP, dBP, infused or non-infused arm BF | No follow-up measurements of AHI or any measure of symptoms; |
| 2008 |  |  | |  | |  | vasodilators used not standard Rx of HTN |
|  |  |  | |  | |  |  |
| Egea | 1 | RCT | | 12 wks | | AHI & ESS ↓ & O2sat ↑in CPAP but not controls; | Only 45 or 60 subjects had OSA; hours/night use not reported; |
| 2008 |  |  | |  | | no other CV parameters changed; | many outcomes but no statistical adjustment for multiple comparisons |
|  |  |  | |  | |  |  |
| Siccoli | 1 | RCT | | 4 wks | | Vs. sham, CPAP ↓ESS (15.8 → 6.8 vs. 15.2 → 11.9, p < 0.001) | # of pts lost to FU not mentioned, despite ITT analysis |
| 2008 |  |  | |  | |  | (disproportionate drop-outs might bias results) |
|  |  |  | |  | |  |  |
| Duran | 2 | RCT | | 12 wks | | CPAP → ↓ 24-h BP (-1.5, p = 0.01), 2) | High drop-out rate (20%; 19% on active Rx), though ITT analysis ↓ risk of bias. |
| 2010 |  |  | |  | | 24-h sBP (-2.1, p = 0.02), & 24-h dBP (-1.3, p = 0.0 | No objective or subjective OSA measures as outcomes |
|  |  |  | |  | |  |  |
| Sharma | 1 | XO | | 12 wks | | Vs. sham, CPAP → ↓ESS (p < 0.001), BMI (-0.29, p < 0.001), | No measure of AHI as an outcome |
| 2011 |  |  | |  | | sBP (-3.9, p = 0.001) |  |
|  |  |  | |  | |  |  |
| **CPAP vs. oral placebo** | | |  | |  | |  |
| Engleman | 2 | XO | | 4 wks | | Vs PBO, CPAP → ↓DT sleepiness (p = 0.03) & overall symptoms (p < 0.001) | Oral placebo & lack of blinding; short follow-up |
| 1994 |  |  | |  | |  |  |
|  |  |  | |  | |  |  |
| Engleman | 2 | XO | | 3 wks | | Overall, CPAP had no effect on DT or NT sBP, dBP or mean aBP; | Small study; short FU; not all patients met criteria for OSA; |
| 1996 |  |  | |  | |  | oral placebo & issue of blinding |
|  |  |  | |  | |  |  |
| Engleman | 2 | XO | | 4 wks | | Vs controls, CPAP ↓symptom score (p < 0.01) & depression score (p = 0.02) | Small study; short FU; poor overall compliance; |
| 1997 |  |  | |  | |  | oral placebo & issue of blinding |
|  |  |  | |  | |  |  |
| Engleman | 2 | XO | | 4 wks | | Vs controls, CPAP → ↑MSLT & ↓ESS & total symptom score (all p ≤ 0.001) | Small study; short FU; poor overall compliance; oral placebo & issue of blinding; |
| 1998 |  |  | |  | |  | treatment order effect identified on analysis secondary to learning |
|  |  |  | |  | |  |  |
| Engleman | 2 | XO | | 4 wks | | Vs controls, CPAP → ↓ total symptoms, ESS & depression (all p < 0.01 | Oral placebo & issue of blinding; poor compliance |
| 1999 |  |  | |  | |  |  |
|  |  |  | |  | |  |  |
| McArdle | 2 | XO | | 4 wks | | Versus PBO, CPAP → ↓ESS, TAI & stage 1 sleep, ↑ stage 3+4 sleep | Small sample; no blinding; oral placebo a questionable control |
| 2000, 2001 |  |  | |  | |  |  |
|  |  |  | |  | |  |  |
| Faccenda | 2 | XO | | 4 wks | | Vs controls, CPAP → ↓ESS, FOSQ, 24-hour dBP | Oral placebo & lack of blinding; short follow-up; |
| 2001 |  |  | |  | |  | no FU of objective OSA or sleep measures (e.g., AHI) |
|  |  |  | |  | |  |  |
| Barnes | 2 | XO | | 8 wks | | CPAP ↓ self-reported OSA Sx vs. placebo, | ? appropriateness of oral placebo, esp. related to blinding; |
| 2002 |  |  | |  | | No improvement in NB fxn, SS* QoL, mood or 24-hr BP | 33% dropout rate; poor CPAP compliance |
|  |  |  | |  | |  |  |
| Barnes | 2 | XO | | 12 wks | | Both splint and CPAP improved Sx vs. placebo (ns) | ? appropriateness of oral placebo, esp. w/ blinding; non-objective |
| 2004 |  |  | |  | |  | measurement of splint compliance; dropouts had worse Sx |
|  |  |  | |  | |  |  |
| **nCPAP vs. conservative care** | | |  | |  | |  |
| Lojander | 2 | RCT | | 52 wks | | VAS daytime somnolence ↓ vs. BL & controls at 12 months (p < 0.05) | Non-blinded; high drop-out rate in CPAP patients |
| 1996 |  |  | |  | |  |  |
|  |  |  | |  | |  |  |
| Redline | 2 | RCT | | 8 wks | | subjects randomized to CPAP (49%) improved vs (26%) controls, | CPAP group may have had increased interactions with Rx team |
| 1998 |  |  | |  | | OR = 2.72 (95% CI: 1.18 - 6.58); |  |
|  |  |  | |  | |  |  |
| Ballester | 2 | RCT | | 12 wks | | ↓sleepiness on ESS; ↑daytime fxn (p < 0.005); ↑energy; ↓social isolation | No blinding; 2 non-validated Q used; no objective measure of OSA; |
| 1999 |  |  | |  | |  | CPAP group lost only 1/3 the wgt of controls (1.1 vs. 3.3 kg) |
|  |  |  | |  | |  |  |
| Monasterio | 2 | RCT | | 24 wks | | Vs controls, CPAP → ↓total symptoms at 3 & 6 months (p < 0.001) | No blinding |
| 2001 |  |  | |  | | No difference in ESS, FOSQ, NHP, MSLT or systolic or diastolic BP |  |
|  |  |  | |  | |  |  |
| Chakravorty | 2 | RCT | | 12 wks | | AHI ↓ in CPAP but not control group; ESS ↓ in both groups; | No blinding; CPAP group had greater access to health care team, |
| 2002 |  |  | |  | |  | which might have biased subjective outcomes like QALY |
|  |  |  | |  | |  |  |
| Kaneko | 2 | RCT | | 4 wks | | CPAP → ↓# apneic/hyponeic episodes (p = 0.002), | Non-blinded; small sample size; short follow-up; mild to no sleep disturbance |
| 2002, 2003 |  |  | |  | | desaturation index (p = 0.008), |  |
|  |  |  | |  | |  |  |
| Mansfield | 2 | RCT | | 12 wks | | Vs controls, CPAP → ↓AHI (p < 0.001), ESS (p = 0.01), | High drop-out rate; small subject sample; non-blinded |
| 2004 |  |  | |  | | ↑ min O2sat (p = 0.001) |  |
|  |  |  | |  | |  |  |
| Drager | 2 | RCT | | 16 wks | | CPAP → ↓ carotid wall thickness, pulse wave velocity, no effect onBP | Only 24/400 pts screened eligible |
| 2006 |  |  | |  | |  | no FU ESS, AHI or other OSA measures |
|  |  |  | |  | |  |  |
| Hsu | 2 | RCT | | 8 wks | | No difference vs. controls in any outcome | Vrey poor CPAP compliance; single blinded; insufficient numbers & power; |
| 2006 |  |  | |  | |  | conservative Rx not described |
|  |  |  | |  | |  |  |
| Lam | 2 | RCT | | 10 wks | | Vs controls, CPAP → ↓AHI, ESS, arousal index, dBP; | Nature of treatments renders blinding of subjects impossible; |
| 2007 |  |  | |  | | ↑ min O2sat & SAQOL index | poor compliance with CPAP (4.2 nights/week) |
|  |  |  | |  | |  |  |
| Barbe | 2 | RCT | | 1 yr | | sBP & dBP ↓ w/ CPAP, but ONLY in pts w/ ≥ 5.65 hr/ngt use | No blinding; no mention of other anti-HTN Rx; BP ↓ only w/ high CPAP use |
| 2010 |  |  | |  | |  | (≥ 5.65 vs. μ 4.7 h/n); no OSA Sx or CV events assessed |
|  |  |  | |  | |  |  |
| Drager | 2 | RCT | | 3 mo | | 24-hr sBP & dBP ↓ vs. control ( p < 0.001). | Small study; non-blinded; no OSA or OSA symptom outcomes |
| 2011 |  |  | |  | | Also ↓ in CPAP vs. control in office sBP, |  |
|  |  |  | |  | |  |  |
| Barbe | 2 | RCT | | med = 4 yr | | Overall rates = 9.20 vs. 11.02 new cases of HTN | Under-powered for an event survey; risk of missed/silent events |
| 2012 |  |  | |  | | per 100 person-years (p = 0.20); |  |
|  |  |  | |  | |  |  |
| **CPAP vs. oral appliance** | | |  | |  | |  |
| Ferguson | 2 | XO | | 2 wks | | CPAP → ↓AHI, apnea index and # of desat. (p < 0.005) & ↑ LSAT (p < 0.05), | Blinding not possible; 2 weeks follow-up; comparisons vs. BL and not between Rx |
| 1996 |  |  | |  | | associated with worse side effects & less satisfaction than OA (both p < 0.05) |  |
|  |  |  | |  | |  |  |
| Ferguson | 2 | XO | | 16 wks | | CPAP →↓AHI, AI, & # desat; ↑LSAT; (odds ratio = 1.91, p = 0.3); | No blinding; no inter-Rx comparisons |
| 1997 |  |  | |  | |  |  |
|  |  |  | |  | |  |  |
|  |  |  | |  | |  |  |
| Engleman | 2 | XO | | 8 wks | | Vs controls, CPAP → ↓AHI, ESS & total symptom score, | No blinding due to different Rx approaches; subjective measure of compliance; |
| 2002 |  |  | |  | |  | multiple comparisons w/ conservative adjustment (p < 0.01) |
|  |  |  | |  | |  |  |
| Randerath | 2 | XO | | 6 wks | | CPAP more effective than OA ↓ AHI (p < 0.01), | Small study (n = 20), short FU (6 wks), no blinding, |
| 2002 |  |  | |  | | snoring (p < 0.01) & ↑ O2sat (p < 0.05); | no validated measure of sleepiness or other OSA symptoms |
|  |  |  | |  | |  |  |
| Tan | 2 | XO | | 8 wks | | CPAP & an MAS ↓AHI (p < 0.001), ESS (p < 0.001), no diff. between Rx. | Small study; no blinding; no compliance data |
| 2002 |  |  | |  | |  |  |
|  |  |  | |  | |  |  |
| Barnes | 2 | XO | | 12 wks | | OA and CPAP improved Sx vs. placebo (ns) | ? appropriateness of oral placebo, esp. w/ blinding; dropouts had worse Sx; |
| 2004 |  |  | |  | |  | non-objective measurement of splint compliance |
|  |  |  | |  | |  |  |
| Lam | 2 | RCT | | 10 wks | | Relative to oral appliance use, CPAP → ↓AHI & ESS (both p < 0.05) | Nature of treatments renders blinding of subjects impossible; |
| 2007 |  |  | |  | |  | OA compliance self-reported |
|  |  |  | |  | |  |  |
| Hoekema | 2 | RCT | | 12 wks | | CPAP & OA → ↓AHI, ESS & %stage 1/2 sleep | High drop-out rate in both Rx arms; no intent-to-treat analysis; no blinding; |
| 2008 |  |  | |  | |  |  |
|  |  |  | |  | |  |  |
| Gagnadoux | 2 | XO | | 8 wks | | CPAP vs. MA, AHI ↓ from median 34 → 2 vs. 6 (inter-group p = 0.001); | Non-blinded; no sham Rx; self-reported compliance for comparison between Rx; |
| 2009 |  |  | |  | | ESS ↓ (NS); | 8 pts unable to use/tolerate MAD |
|  |  |  | |  | |  |  |
| Trzepizur | 2 | XO | | 8 wks | | Both CPAP & MAD ↓ AHI (p < 0.05) but not ESS (11 → 10 vs. 9, NS) | Non-blinded; only 12 patients in cross-over; almost 30% dropout rate |
| 2009 |  |  | |  | |  |  |
|  |  |  | |  | |  |  |
| Aarab | 2 | RCT | | 6 mo | | AHI ↓w/ CPAP (p < 0.001) & MAD (p < 0.001) | > 1 in 4 sham treated pts knew they were on sham. CPAP drop-out rate ~ 20%; |
| 2011 |  |  | |  | | vs sham (20.1 → 14.9) (p < 0.001 ) | hrs/ngt of CPAP & MAD use not reported; can't truly blind CPAP vs. OA |
|  |  |  | |  | |  |  |
| Aarab | 4 | parallel | | 1 yr | | Benefits of nCPAP & MAD maintained at 6 & 12 months | High drop out rate in CPAP group; no compliance, non-blinded; no ITT analysis |
| 2012 |  | cohorts | |  | |  |  |
|  |  |  | |  | |  |  |
| Hoekema | 4 | parallel | | ~2 yrs | | Both CPAP & OA → ↓ AHI, ESS; ↑ FOSQ scores | Original cross-over study only published as an abstract; |
| 2013 |  | cohorts | |  | |  | hence, no verifiable data from original study |
|  |  |  | |  | |  |  |
| **CPAP vs. posture Rx** | | |  | |  | |  |
| Jokic | 2 | XO | | 2 wks | | Vs postural device, CPAP → greater ↓ in AHI (p = 0.007) | Single blind only; small subject same; short follow-up |
| 1999 |  |  | |  | | ↑ in LSAT (net rise 4.0%; p = 0.02). |  |
|  |  |  | |  | |  |  |
| Skinner | 2 | XO | | 4 wks | | On CPAP, 12/14 were complete responders, | Small study; non-blinded; short FU; somewhat arbitrary definition of |
| 2004 |  |  | |  | | 1/14 partial & 1/14 non-compliant; | response, partial response, non-response |
|  |  |  | |  | |  |  |
| Skinner | 2 | XO | | 4 wks | | On CPAP, 7/10 complete, 1/10 partial & 2/10 non-responders; | Small study; non-blinded; short FU; somewhat arbitrary definition of |
| 2004 |  |  | |  | | w/ support collar (p < 0.05); | response, partial response, non-response |
|  |  |  | |  | |  |  |
| Permut | 1 | XO | | 1 night | |  | One night FU; non-blinded; CPAP compliance not reported |
| 2010 |  |  | |  | | No ∆ in sleep efficieny or architecture or arousal index. |  |
|  |  |  | |  | | 50% vs. 34% preferred the PD. |  |
| **CPAP alone - compliance** | | |  | |  | |  |
| Engelman | 2 | cohort | | 12 wks | | Mean use of CPAP was 4.7 hours/night; | No control group; only outcome of interest = compliance |
| 1994 |  |  | |  | | therapeutic pressure was achieved 89% of the time; |  |
|  |  |  | |  | |  |  |
| Sharma | 1 | XO | | 1 night | |  | One night of observation only; unable to assess compliance in sleep lab |
| 1996 |  |  | |  | | Final CPAP = 12.3 vs. 10.1 mmH20 (p < 0.05) |  |
|  |  |  | |  | |  |  |
| Jenkinson | 2 | cohort | | 6 mo | | Initial gains in Rx group were maintained, | Non-blinded cohort study; no objective measures of OSA severity at FU |
| 2001 |  |  | |  | | while those switching to nCPAP achieved similar gains |  |
|  |  |  | |  | |  |  |
| Ballard | 1 | RCT | | 3 mo | | After 90 days, improved compliance achieved in 25/51 (49%) | Only short term data studied |
| 2007 |  |  | |  | |  |  |
|  |  |  | |  | |  |  |
| Weaver | 3 | cohort | | 3 mo | | In pts using CPAP 4-5 h/n, (mean use = 4.7 h/n), | Non-controlled study |
| 2007 |  |  | |  | | ESS normalization was achieved in 73.3% |  |
|  |  |  | |  | |  |  |
| Marshal | 1 | RCT | | 4 wks | | 1.7 h/n more use in Cflex group but not statistically significant | Very small study, under-powered to identify > 55% difference |
| 2008 |  |  | |  | |  | in the primary outcome |
|  |  |  | |  | |  |  |
| To | 1 | XO | | 2 mo | | At 1 & 2 mo., auto CPAP was used more in total (p < 0.05) | Non-blinded study; technical limitations of the fixed device |
| 2008 |  |  | |  | |  | preclude assessment of residual AHI |
|  |  |  | |  | |  |  |
| Wolkove | 3 | survey | | μ = 64 mo | | Of 80 initially on CPAP, | No verification of CPAP use; all data by self-report; |
| 2008 |  |  | |  | | 25 stopped it right after titration & 12 more within a mean 10.1 mo. | no objective measure of OSA severity at FU |
|  |  |  | |  | |  |  |
| Bakker | 1 | RCT | | 12 wks | | MSLT better with CPAP at 3 months, but not at 1 month | Only powered to detect a 1.3hr/night mean difference in device use |
| 2009 |  |  | |  | |  |  |
|  |  |  | |  | |  |  |
| Ruhle | 1 | XO | | 4 wks | | More nasal and oropharyngeal side effects w/ std. CPAP (p < 0.05); | How compliance was measured & recorded not mentioned |
| 2009 |  |  | |  | | non-significant ↑ in compliance |  |
|  |  |  | |  | |  |  |
| Ryan | 1 | RCT | | 4 wks | | ESS ↓ from 14 → 5, p < 0.001. Similar efficacy w/ all 3 Rx | Unable to objectively measure steroid and humidifier use (self-reported only) |
| 2009 |  |  | |  | |  |  |
|  |  |  | |  | |  |  |
| Roecklein | 1 | RCT | | 3 mo | | No significant difference between groups | No analysis to assess how compliance affects OSA severity or symptoms |
| 2010 |  |  | |  | |  |  |
|  |  |  | |  | |  |  |
| Venelle | 1 | XO | | 6 wks | | ESS lower (p = 0.03) and compliance higher (p = 0.047) | 10% drop-out rate; short FU |
| 2010 |  |  | |  | | with variable vs. fixed pressure CPAP |  |
|  |  |  | |  | |  |  |
| Kohler | 1 | RCT | | 2 wks | | No change in psychomotor fxn, inflam. markers, lipids or insulin res. | Very short-term study |
| 2011 |  |  | |  | |  | thereby influencing subjective outcomes |
|  |  |  | |  | |  |  |
| Kryger | 4 | cohort | | 1 yr | | In responders with compliance ≥ 5h/n from an earlier RCT (EPAP vs. sham), | Subjects = prior responders w/ good compliance; no controls; open label; |
| 2011 |  |  | |  | |  | adherence by self-report; strict exclusion criteria for initial RCT |
|  |  |  | |  | |  |  |
| Kushida | 1 | RCT | | 6 mo | | AHI & MSAT higher for Aflex immediately after titration, | No sham control |
| 2011 |  |  | |  | | but all differences lost by 6 months. |  |
