# Supplementary material for: Reconsidering first-line treatment for obstructive sleep apnea: a systematic review of the literature
Source: J Otolaryngol Head Neck Surg. 2016 Apr 6;45:23. doi: 10.1186/s40463-016-0136-4 (PMC4822285; doi:10.1186/s40463-016-0136-4)
Supplement: Additional file 2: Table S2. — Surgery findings. (DOCX 43 kb) [file 40463_2016_136_MOESM2_ESM.docx]

Table S2: Surgery findings

| First author | EBM | Study | Study | Study |
| --- | --- | --- | --- | --- |
| year | rating | design | findings | limitations/issues |
| **UPPP, UP Flaps +/- Other** | | |  |  |
| Djupesland | 4 | CS | AHI ↓(p < 0.001); 10/20 ↓AHI > 50%; 18/20 reported immediate post-op ↓ in DT sleepiness | Non-controlled study; small sample; no validated measures |
| 1992 |  |  | sBP ↓from 154 → 142 (p < 0.05); no change in dBP or MAP; weight ↓ from 96.6 → 87.8kg | of sleepiness, snoring or other OSA symptoms |
|  |  |  |  |  |
| Johnson | 4 | prosp | In CPAP failures, RDI ↓(58.7 → 14.5, p < 0.001) | Non-controlled study; very small sample; no validated measures |
| 1994 |  | CS |  | of sleepiness, snoring or other OSA symptoms |
|  |  |  |  |  |
| Lojander | 2 | RCT | # O2-desat. events ≥ 4% (ODI_4_) & ≥ 10% (ODI_10_) ↓ post surgery 12 months FU (p < 0.001); | Non-blinded; can't compare CPAP & surgery patients |
| 1996 |  |  |  | because surgical candidates not randomized to CPAP |
|  |  |  |  |  |
| Ramirez | 4 | prosp | Post-op, while still on nCPAP, RDI ↓(49.0 → 23.0, p < 0.001) | Non-controlled study; very small sample; no validated measures |
| 1996 |  | CS |  | of sleepiness, snoring or other OSA symptoms |
|  |  |  |  |  |
| Michelson | 4 | CS | No statistically significant change in AHI, RDI or lowest O2sat | Very small sample, non-random, non-controlled, non-blinded, variable FU |
| 1997 |  |  |  |  |
|  |  |  |  |  |
| Elasfour | 3 | prosp | AHI ↓ from 52.6 → 23.9 & LSAT ↑ from 73.8 → 85.5% (p < 0.001); | No non-surgical control; non-random allocation w/ potential selection bias; |
| 1998 |  | CC | In UPPP + TBR pts, AHI ↓ from 65.0 → 29.2 & LSAT ↑ from 71.7 → 81.9% | small numbers; no measure of OSA symptoms |
|  |  |  |  |  |
| Lee | 4 | prosp | AHI ↓(53 → 19, p < 0.001); all responders reported complete resolution of snoring. | Non-controlled study; no actual data provided on OSA symptoms |
| 1999 |  | CS |  | (despite improvements in responders) |
|  |  |  |  |  |
| Wilhelmsson | 2 | RCT | AHI normalized at 12 mo in 51 vs. 78% (p < 0.05) | no ITT analysis; 25% d/o rate in OA group |
| 1999 |  |  |  | post randomization; UPPP group worse at BL |
|  |  |  |  |  |
| Walker-Engstrom | 2 | RCT | QoL scale improved vs. baseline post UPPP vs. 10/24 with OA. | BL & FU polysomnography scores not reported; non-blinded; |
| 2000 |  |  |  | non-validated sleep measure; MSE-P uncommonly used (only 18 medline ref) |
|  |  |  |  |  |
| Hendler | 4 | retro | AHI ↓ from 60.2 → 28.8 (p < 0.001); LSAT ↑ from 72.7 → 80.4% (p = 0.02) | Non-controlled study; no measure of OSA symptoms |
| 2001 |  | CS |  |  |
|  |  |  |  |  |
| Hendler | 4 | retro | AHI ↓ from 90.1 → 16.5, (p < 0.001); LSAT ↑ from 64.9 → 88.2%% (p = 0.009) | Non-controlled study; very small sample; no measure of OSA symptoms |
| 2001 |  | CS |  |  |
|  |  |  |  |  |
| Nelson | 4 | prosp | UPPP + TC-RFTR, AHI ↓(29.5 → 18.8, p = 0.07); | No non-surgical controls; very small samples, insufficient power; |
| 2001 |  | CC |  | different severity OSA in 2 groups; non-random group allocation |
|  |  |  |  |  |
| Terris | 4 | retro | AHI ↓from 32.4 → 14.4 (p < 0.01) & ESS ↓from 11.0 → 5.4 (p < 0.005). | Non-random, non-controlled, non-blinded restrospective series w/ unknown FU & |
| 2002 |  | CS |  | > 1/3 drop-out rate; analysis biased by only assessing responders |
|  |  |  |  |  |
| Vilaseca | 4 | prosp | Those with AHI ≤ 60 had AHI < 20 & symptoms 'significantly improved' at 6 months | Non-random, non-controlled small series w/ non-blinded assessments |
| 2002 |  | CS |  |  |
|  |  |  |  |  |
| Walker | 2 | RCT | 30 of 40 (75%) post UPPP required no further treatment; | High drop-out rate (~ 25%); no blinding; potential for unrecorded contamination |
| 2002 |  |  |  | over 4 years FU (postural changes, etc.) |
|  |  |  |  |  |
| Cahali | 4 | prosp | AHI ↓ from 41.2 → 9.5 (p = 0.009) | 10 other pts randomized to UPPP, but this group not reported; small sample; non-blinded |
| 2003 |  | CS |  |  |
|  |  |  |  |  |
| Friedman | 4 | retro | AHI ↓(43.9 → 28.1, p < 0.05) w/ UPPP + RFTR, vs. AHI ↓(35.4 → 26.5, p < 0.05) w/ UPPP alone. | No non-surgery controls; non-random allocation: |
| 2003 |  | CC |  | UPPP+ procedures since 2000 |
|  |  |  |  |  |
| Neruntarat | 4 | prosp | RDI ↓(44.5 → 15.2, p < 0.001); 78.0% 'success rate'; LSAT ↑(82.1 → 87.9, p < 0.01); | Non-controlled study |
| 2003 |  | CS | ESS ↓ (14.1 → 8.2, p < 0.01); snoring VAS ↓ (8.5 → 3.5, p < 0.001) |  |
|  |  |  |  |  |
| Neruntarat | 4 | prosp | AHI ↓(47.9 → 14.2 → 18.6, p < 0.001); LSAT ↑(81.2 → 88.8 → 87.2%, p < 0.01); | Non-controlled study |
| 2003 |  | CS | ESS ↓ (15.9 → 6.2 → 7.3, p < 0.01); snoring VAS ↓ (8.5 → 1.7 → 5.2, p < 0.01) |  |
|  |  |  |  |  |
| Cahali | 1 | RCT | Both significantly ↓ ESS, VAS snoring, DT sleepiness, AM headaches | Small sample; no blinding of pts mentioned, which MIGHT influence |
| 2004 |  |  |  | self-report measures |
|  |  |  |  |  |
| Dattilo | 4 | CS | 14/15 treatment successes | Non-controlled study |
| 2004 |  |  | ESS ↓ from 14.5 →7.5 & from 17.8 → 4.7 (both p < 0.001). |  |
|  |  |  |  |  |
| Li | 4 | retro | 44/55 = Rx success with RDI ↓>50% to < 20; mean RDI ↓ (43.6 → 12.1), ESS ↓(11.8 → 7.5) | Non-controlled; retrospective data collection; |
| 2004 |  | CS | LSAT ↑ (78.9 → 84.6) - all p < 0.001. | definition of success less stringent than others (RDI < 20) |
|  |  |  |  |  |
| Miller | 4 | retro | RDI ↓(52.9 → 15.9, p < 0.001); LSAT ↑ 80.0 → 88.0%, p = 0.002); | Non-controlled study; > 30% of original sample not assessed post-op; |
| 2004 |  | CS | posterior airway ↑ 7.9 → 12.6 mm, p < 0.0001) | no OSA symptom outcomes |
|  |  |  |  |  |
| Weaver | 4 | prosp | At both 3 & 6 mo, ↓ESS (12.9→7.0→6.9); | non-controlled; 51% dropout rate @ 3 mo, 50% @ 6 mo |
| 2004 |  | CS | FOSQ (14.3, 17.2, 17.5) (all p < 0.001 except HA - p = 0.048 & 0.008) |  |
|  |  |  |  |  |
| Yu | 3 | nonRCT | in group 1, RDI ↓ from 50.7 → 8.0 (p < 0.01) & LSAT↑ from 76.3 → 88.3% (p = 0.06) | No non-surgical control; non-random allocation w/ potential selection bias; |
| 2004 |  |  |  | very small numbers; no measure of OSA symptoms |
|  |  |  |  |  |
| Verse | 3 | nonRCT | With hyoid suspension, AHI ↓(38.9 → 20.7, p < 0.001), | Non-random allocation to group w/ potential slection bias; |
| 2006 |  |  | LSAT, MSAT & arousal index ↑ (all p < 0.05) & ESS ↓ (p < 0.05). | Group A had more severe OSA (AHI = 38.9 vs. 27.8) & shorter FU (4.3 vs. 5.9 mos) |
|  |  |  |  |  |
| Pang | 4 | props | In the 39 with OSA, AHI ↓from 25.3 → 11.0 & LSAT ↑ from 81.4 → 92.0 (both p < 0.05). | Non-controlled study; few outcomes |
| 2009 |  | CS |  |  |
|  |  |  |  |  |
| Yu | 4 | retro | UPPP → ↓AHI (42.5→14.5), ↑LSAT (72.7→86.6%). Also → ↓ 24-hr sBP & dBP | Non-controlled study; short FU (1 mo) |
| 2010 |  | CS |  |  |
|  |  |  |  |  |
| Aneeza | 4 | retro | In 10 pts w/ post-op AHI, median ↓ from 52 → 31 (p = 0.16); | Post-op AHI data missing for ~ 75% & ESS data for 63% of cases; non-controlled; |
| 2011 |  | CS |  | non-blinded assessments; extremely variable length of FU |
|  |  |  |  |  |
| Neruntarat | 4 | prosp | VAS snoring rating: 8.6, 2.4 (p < 0.001), 3.2 (p < 0.01). 78% short-term, 52% long-term 'success' | Non-controlled study; highly variable time of final FU (48 - 62 mo.) |
| 2011 |  | CS |  |  |
|  |  |  |  |  |
| Weaver | 1 | pop. | 1339/18754 (7.1%) died w/ CPAP vs. 71/2072 (3.4%) post-op. | Retrospective analysis; potential confounders missing |
| 2011 |  | survey | Adjusting for covariates, mortality ↑ 31% (95% CI 3 - 67%) w/ CPAP (p = 0.03) | (e.g., severity of OSA, overall health status) |
|  |  |  |  |  |
| Yaremchuk | 4 | retro | ESS ↓ from 11.1 → 5.5 (p = 0.001); AHI ↓ from 36.1 → 13.0 (p = 0.001), | ESS data unavailable for 36% & AHI data for 545 of the initial sample; |
| 2011 |  | CS |  | non-controlled; retrospective data collection |
|  |  |  |  |  |
| Lee | 4 | prosp | AHI ↓ from 55.6 → 24.1 (p < 0.001); LSAT ↑ from 75.8 → 81.7% (p = 0.01); | Non-controlled study; highly variable time of final FU (4 - 19 mo.) |
| 2012 |  | CS | ESS ↓ from 13.4 → 5.9 (p = 0.003). In 9/20, AHI ↓ ≥50% to < 20; in 13/20, AHI ↓ ≥50% |  |
|  |  |  |  |  |
| Yang | 3 | prosp | AHI ↓ (46.5 → 8.8), LSAT ↑ (69.1 → 81.2%), & MSAT ↑(82.6 → 93.4%), all p < 0.05. | No 'sham-treated' controls; no measure of OSA symptoms at BL or FU |
| 2012 |  | CC |  |  |
|  |  |  |  |  |
| Baugh | 2 | pop. | 94% OF nasal, 86% of palatal & 79% opf combined nasal+palatal surgeries were ambulatory | Administrative data set not all-inclusive; |
| 2013 |  | survey |  | pts from other states or who moved may have FU data missed |
|  |  |  |  |  |
| Mackay | 4 | prosp | AHI ↓from 23.1 → 5.6; ESS ↓ from 10.5 → 5.0; & LSAT ↑ from 86.0 → 89.5% (all p < 0.05) | Non-controlled study; few outcomes; |
| 2013 |  | CS |  | no objective measure of sleepiness; no function or QoL outcomes |
|  |  |  |  |  |
| Rotenberg | 2 | prosp | AHI ↓ compared to CPAP; p < 0.001; ESS ↓ (14.3 → 4.1, vs. 13.7 on CPAP; p < 0.001 vs. BL); | Historic vs. current CPAP controls; non-blinded; |
| 2014 |  | cohort | sBP ↓ (143.2 → 134.5, vs. 144.3 on CPAP; p < 0.001) | 11 pts lost to FU; no ITT analysis |
|  |  |  |  |  |
| Baradaranfar | 4 | prosp | AHI ↓(23.2 → 10.5, p < 0.001); RDI ↓ (26.4 → 9.4, p < 0.001);LSAT ↑ (78.8 → 81.8, p < 0.001); | Non-controlled study; unclear if study started w/ 54 or 48 pts (? 6 drop-outs); |
| 2015 |  | CS | ESS ↓ (12.6 → 7.1, p < 0.001) | clinically insignificant ↑ in MSAT (though statistically significant) |
|  |  |  |  |  |
| **Mandibular Advancement** | | |  |  |
| Bettega | 4 | prosp | AHI ↓ 45.2 → 42.8 (NS), | Non-controlled study |
| 2000 |  | CS | (75% success). μSAT & LSAT ↑ and time w/ SAT < 90% ↓(64→5%), all p < 0.01 | (? selection bias); no OSA symptoms as outcomes |
|  |  |  |  |  |
| Li | 4 | CS | > 6 months post-op, RDI ↓from 63.6 → 8.1 (p < 0.001) & LSAT ↑ from 73.3 → 88.1% (p < 0.01) | Very small sample, non-random, non-controlled, non-blinded, variable FU |
| 2000 |  |  |  |  |
|  |  |  |  |  |
| **Laser Treatments** | | |  |  |
| Michelson | 4 | CS | AHI ↓ (19.4 → 4.2, p = 0.006), RDI ↓ (31.2 → 15.7, p = 0.09) | Non-random, non-controlled, non-blinded with extremely variable FU |
| 1996 |  |  | & snoring ↓ in 12/13 (p < 0.001) | & almost 2/3 drop-out rate |
|  |  |  |  |  |
| Walker | 4 | retro | Among 15 w/ mild OSA, RDI ↓ in 9, no ∆ in 3 & ↑ in 3; among 12 w/ moderate OSA, 7 ↓, 1 no ∆ & 4 ↑; in 11 w/ severe ODA, 7 ↓, 3 no ∆, 1 ↑. | Small, non-controlled study. Insufficient power for group comparisons |
| 1999 |  | CS | Overall, RDI ↓in 23, no ∆ in 7, ↑ in 8. Rx success in 46.7, 41.7 & 45.5% | (by OSA severity) |
|  |  |  |  |  |
| Finkelstein | 4 | CS | At final FU, 50% improved, 35% unchanged, 15% worse; 31% had successful | Small study, no controls, no measure of sleepiness |
| 2002 |  |  | ↓ in RDI (50%↓ or < 20). Overall 58% satisfied, 42% dissatisfied. | pts asked to rate their own snoring (how would they know?) |
|  |  |  |  |  |
| Ferguson | 2 | RCT | Vs. controls, AHI ↓ (from 18.8 → 14.7, p = 0.04); but no difference in ESS or SAQLI. | No blinding; bias related to clear intervention vs. clear non-intervention |
| 2003 |  |  | 52% were satisfied, 48% dissatisfied w/ the surgery |  |
|  |  |  |  |  |
| Kyrmizakis | 4 | prosp | At 3-4 mo FU, 15/25 pts (60%) had AHI ↓ to ≤ 5; 8 no to little change, | Non-controlled, small study; few outcome measures; no long-term repeat |
| 2003 |  | CS |  | of objective OSA measures; no validated questionnaires |
|  |  |  |  |  |
| Atef | 1 | RCT | LAUP after 1-5 Rx ↓ AHI from 25.5 → 15.9 11.5, 9.7, 9.0 & 8.8 after 2, 3, 4 & 5 Rx at 3 mo FU | No mention of drop-outs. Paper has VAS as a second outcome but never says what |
| 2005 |  |  |  | the VAS is measuring (? Snoring?) |
|  |  |  |  |  |
| Lin | 3 | CC | Responders experienced dramatic improvements in RDI (38.3 → 9.4), | Non-random, small study. Selective analysis of responders questionable; |
| 2006 |  |  | LSAT (69.6 → 90.5%), sleep architecture | no baseline characteristic distingushed responders vs. non-responders |
|  |  |  |  |  |
| **Radiofrequency/Other Tongue Procedures** | | |  |  |
| Powell | 4 | prosp | After a mean 5.5 treatments, ↓ RDI (39.6 → 17.7, p = 0.03); ↑ LSAT (81.9 → 88.3%, p = 0.03); | Inadequate power to detect QoL differences vs. pre-Rx. |
| 1999 |  | CS |  | Two treated pts had baseline RDI < 1.5 (why were they treated for OSA?) |
|  |  |  |  |  |
| Woodson | 4 | CS | ↓ AHI (23.8→16.3, p < 0.01), ESS (13.8→8.8, p = 0.002), VAS sleepiness (5.8→8.8, p = 0.01), | A parallel group (n = 14) were snorers w/o OSA, but no true controls; |
| 2001 |  |  |  | non-random; 33% drop-out rate |
|  |  |  |  |  |
| Woodson | 4 | 2 cohorts | AHI ↓ from 40.5 → 32.8 (p < 0.01) No ∆ in LSAT. ESS ↓from 11.8 → 7.4 post-RFTR | Non-random cohorts drawn from separate studies; 9 centres (5 non-academic) |
| 2001 |  | compared |  | from across USA (consistentcy in data collection?); lots of missing data |
|  |  |  |  |  |
| Riley | 4 | prosp | ↓AHI (35.1 → 15.1, p < 0.001), ↑LSAT (82.0 → 86.3%, p < 0.01), | Non-controlled; |
| 2003 |  | CS |  | for changes if compliance vs. pre-op increased) |
|  |  |  |  |  |
| Thomas | 1 | RCT | Both Rx ↓ ESS (TA: 13.4 →5.4, p = 0.004; TS: 12.1 → 4.1, p = 0.007; NS diff.) | Very small sample; non-blind assessments |
| 2003 |  |  |  |  |
|  |  |  |  |  |
| Woodson | 1 | RCT | Relative to sham Rx, reaction time improved post-RFTR (p = 0.03 & 0.02) but not on CPAP. | Very poor CPAP complance (~ 16 hours/week); |
| 2003 |  |  |  | different # of Rx sessions in RFTR (4.5) vs. sham RFTR (2.9) groups |
|  |  |  |  |  |
| Stuck | 4 | prosp | RDI ↓ from 25.3 → 16.7 (p < 0.5) snoring ↓from 7.4 → 3.7 on 10cm VAS (p < 0.05). | Non-controlled; definition of 'cure' ignored all symptoms |
| 2004 |  | CS |  |  |
|  |  |  |  |  |
| Atef | 1 | RCT | See above under Laser Treatments | See above under Laser Treatments |
| 2005 |  |  |  | the VAS is measuring (? Snoring?) |
|  |  |  |  |  |
| Steward | 4 | prosp | ↓AHI (19.0 → 8.6, p = 0.01), ESS (12.9 → 9.5, p < 0.001), | FU AHI only available for 20/29 pts; extremely variable FU; |
| 2005 |  | CS | SNORE25 (p < 0.001), median reaction time (p = 0.03) & ↑ FOSQ (p < 0.001) | non-random, non-blinded |
|  |  |  |  |  |
| Bassiouny | 1 | RCT | AHI ↓ from 17.2 → 8.1 vs. 15.3 → 9.8 (both p < 0.001; no inter-Rx diff.); | Small study; few outcomes; no sleep, function or QoL assessment |
| 2007 |  |  | VAS snoring ↓from 7.5 → 5.5 ( p < 0.001; no inter-Rx diff.) |  |
|  |  |  |  |  |
| Ceylan | 3 | nonRCT | Both RFTR & CPAP →↓AHI (28.5→15.7 vs 29.6→16.1, both p < 0.001; NS); | Non-random allocation to Rx/ potential selection bias; |
| 2009 |  |  | ↓ESS (11.1→8.4, p = 0.003 vs 10.8→8.2, p = 0.003; NS); | compliance with CPAP not reported |
|  |  |  |  |  |
| **Palate Implants/Procedures** | | |  |  |
| Norgaard | 4 | prosp | AHI ↓ from 16.2 → 12.1 (p < 0.05), with 12/25 (44%) → AHI ≤ 10. | Non-controlled, small study. |
| 2006 |  | CS | ESS ↓ from 9.7 → 5.5 (p < 0.001) |  |
|  |  |  |  |  |
| Walker | 4 | CS | AHI ↓but just from 25.0 → 22.0 (p = 0.05). | Non-controlled study; few outcomes, not clinically signiciant |
| 2006 |  |  | Mean ESS ↓ from 11.0 → 6.9 & 100 MM snore rating ↓ from 7.9 → 4.0 (both p < 0.001) |  |
|  |  |  |  |  |
| Pang | 4 | CS | AHI ↓ from 12.3 → 5.2 (p < 0.05), ESS ↓ from 12.2 → 8.9 & LSAT ↑ from 88.3 → 92.5 (p < 0.05). | Very small study; non-controlled; few outcomes; |
| 2007 |  |  | Snoring also improved (from 8.3 → 3.3 including 5 non-OSA pts w/ snoring alone; p < 0.05) | no objective measures of sleep or of QoL |
|  |  |  |  |  |
| Friedman | 1 | RCT | AHI ↓ (23.8 → 15.9 vs. 20.1 → 21.0, p < 0.0001); ESS ↓ (12.7 → 10.2 vs. 11.7 → 11.1, p < 0.05); | Imbalanced drop-outs (2 active, 5 sham) may bias ITT analysis |
| 2008 |  |  |  | w/ dropouts rated Rx 'failures"; sham group 9 years younger |
|  |  |  |  |  |
| Back | 1 | RCT | ESS ↓ from 10.0 → 8.0 vs. 8.0 → 5.0 (NS); AHI ↑ from 11.0 → 13.0; | Very small sample; very mild OSA |
| 2009 |  |  | no change at all in LSAT, MSAT or compound endpoint score for either Rx |  |
|  |  |  |  |  |
| Huang | 3 | nonRCT | AHI ↓(14.1 → 9.0 vs. 14.2 → 8.8 vs. 14.1 → 6.1, p < 0.05); | Small study, non-blinded study w/ no non-surgical controls |
| 2011 |  |  | LSAT ↑ (86.2 → 90.0; 91.0 → 92.0; 89.1 → 91.1, NS) |  |
|  |  |  |  |  |
| **SAFETY** | | |  |  |
| Kerizian | 2 | prosp | Very low rate of respiratory and other non-fatal complications | VA National Surgical Quality Improvement Program (NSQIP) database |
| 2004 |  | cohort | haemorrhage 0.3%. 30-day mortality = 0.2% | limited in data collected; potential for coding errors |
|  |  |  |  |  |
| Strocker | 4 | retro | 90% discharged home same day; | Retrospective data collection; few details provided; |
| 2008 |  | cohort | no complications or rehospitalizations over the next 3-4 weeks | no outcomes beyond time of discharge & major complications |
|  |  |  |  |  |
| Rotenberg | 2 | prosp | Of 121 pts, only 4 required significant nursing intervention: | Nursing intervention guidelines ≠ # desaturations; |
| 2010 |  | cohort | 3 required supplemental CPAP, 1 supplemental O2 + CPAP; 3/4 had had multilevel surgeries. | insufficient power to detect predictors |
|  |  |  |  |  |
| Pang | 2 | retro | Overall complication rate = 1.0%: 15 w/ persistent post-op HTN; 15 secondary bleeding; | Incompleteness of hospital records; no pre-defined criteria |
| 2012 |  | cohort |  | (e.g., for desaturation); ascertainment bias |
|  |  |  |  |  |
| Kandasamy | 2 | retro | Comparing outpt vs. inpt cases: total complication rate = 21.6 vs. 33.9% (p = 0.03); | Retrospective data collection; |
| 2013 |  | cohort |  | LSAT data often unavailable |
|  |  |  |  |  |
